# *sepal*: identifying transcript profiles with spatial patterns by diffusion-based modeling

**DOI:** 10.1093/bioinformatics/btab164

**Published:** 2021-03-11

**Authors:** Alma Andersson, Joakim Lundeberg

**Affiliations:** Department of Gene Technology, Science for Life Laboratory, KTH Royal Institute of Technology, Stockholm 114 28, Sweden; Department of Gene Technology, Science for Life Laboratory, KTH Royal Institute of Technology, Stockholm 114 28, Sweden

## Abstract

**Motivation:**

Collection of spatial signals in large numbers has become a routine task in multiple omics-fields, but parsing of these rich datasets still pose certain challenges. In whole or near-full transcriptome spatial techniques, spurious expression profiles are intermixed with those exhibiting an organized structure. To distinguish profiles with spatial patterns from the background noise, a metric that enables quantification of spatial structure is desirable. Current methods designed for similar purposes tend to be built around a framework of statistical hypothesis testing, hence we were compelled to explore a fundamentally different strategy.

**Results:**

We propose an unexplored approach to analyze spatial transcriptomics data, simulating diffusion of individual transcripts to extract genes with spatial patterns. The method performed as expected when presented with synthetic data. When applied to real data, it identified genes with distinct spatial profiles, involved in key biological processes or characteristic for certain cell types. Compared to existing methods, ours seemed to be less informed by the genes’ expression levels and showed better time performance when run with multiple cores.

**Availabilityand implementation:**

Open-source Python package with a command line interface (CLI), freely available at https://github.com/almaan/sepal under an MIT licence. A mirror of the GitHub repository can be found at Zenodo, doi: 10.5281/zenodo.4573237.

**Supplementary information:**

Supplementary data are available at *Bioinformatics* online.

## 1 Introduction

It has become evident in the post-NGS (Next Generation Sequencing) era that biological systems are best understood when studied in their entirety, as the intricate interplay between their constituents is easily lost if these parts are examined in isolation. In the field of transcriptomics this idea, the importance of context, has largely equated to examining the expression of multiple genes simultaneously, either on bulk or single cell level. However, there has been a recent surge in the development of experimental techniques designed to obtain *spatial* gene expression data, completely redefining the meaning of context. Armed with these techniques researchers are no longer limited to only quantifying expression levels, but may also relate them to each other in the spatial space. Different approaches to obtain spatial gene expression data have been presented: some, like ISS (In Situ Sequencing), rely on predesigned probes or gene panels; others aim for unbiased sampling from the whole transcriptome, for example by capture of polyadenylated mRNA molecules as in Visium, Spatial Transcriptomics (ST) and Slide-seq (Ke *et al.*, 2013; Rodriques *et al.*, 2019; Ståhl *et al.*, 2016). One benefit of targeting the whole transcription landscape is how a more representative portrait of the system’s state is obtained. Still, with a less specific set of targets, additional noise is unavoidably introduced into the data. Techniques designed to capture the full transcriptome may therefore be considered as better suited for exploratory analysis compared to those with *a priori* target selection, but also demand more elaborate processing to distill significant signals from the background noise.

For this reason, gene expression profiles that possess distinct spatial patterns are of particular interest when attempting to chart the biological processes and pathways present within a tissue using transcriptome-wide techniques; structured spatial organization is unlikely to arise spontaneously, rather it implies presence of an underlying mechanism driving the system toward the observed configuration. Motivated by this, we have developed a method designed to analyze full transcriptome spatial data and extract gene expression profiles that exhibit distinct spatial patterns.

Humans can intuitively detect deviances or features with captivating characteristics in spatial data; the challenge lies in translating such intuition into an automated and unbiased method suitable for computational analysis. To start, spatial patterns are diverse and do not follow a single well-defined distribution. In addition, most experimental techniques do not collect data continuously from the spatial domain, but sample from this space with varying sparsity; meaning that the complete spatial expression profiles are rarely observed. To address these issues, existing methods have either used non-parametric tests based on permutation of data (Trendsceek) or tried to model the generative process producing the spatial data to then determine whether a spatial effect is present (SpatialDE and SPARK) (Edsgärd *et al.*, 2016; Sun *et al.*, 2020; Svensson *et al.*, 2018).

In this study, we seek to explore a different strategy not centered around hypothesis testing, with the intention to present complementary insights to those offered by other methods. Thus, we abstain from any attempt to infer or characterize the exact distributions from which the observed data originates, instead, we seek to assess the degree of randomness exhibited by each transcript profile and rank them accordingly. Upon doing so, we consider transcripts with a spatially random distribution as antipodes to those with distinct spatial patterns. To locate where on the spectrum between random and structured that a certain gene expression profile is positioned, we simulate diffusion of transcripts in the spatial domain and measure the time until convergence. The profiles are then ranked by this value, with the rationale being that transcripts with a random spatial distribution will reach a homogeneous state faster than those with a structured formation. To elaborate slightly on this idea; if transcripts of a certain gene (theoretically) were let to diffuse freely within a tissue, more time would be required to even out the concentration gradients present in a structured pattern compared to a more uniform state. This reasoning links the degree of structure among transcripts to the time it takes the system to reach a homogeneous configuration, and also implies a positive correlation between the two. Hence, by measuring the diffusion time in our simulated system we would be able to infer how structured or ‘non-random’ the expression pattern in question is. This, to our knowledge, is an unexplored strategy for identification of spatial gene expression patterns.

After the gene expression profiles have been ranked, we suggest a procedure to group them into pattern families, where members of the same pattern family exhibit similar spatial organization. This assortment encompass the construction of an eigenpattern space onto which the gene expression profiles are projected and then hierarchically grouped.

While this study mainly focuses on array-based spatial transcriptomics techniques such as ST and Visium, we also show how our method can be generalized to platforms where the spatial positions of observations do not adhere to a fixed arrangement (like that of an array), but vary between experiments. We refer to data collected from these platforms as *unstructured*, since distances between observations and their relative positions are random and do not adhere to a pre-defined structure. Examples of platforms producing unstructured data are: Slide-seq, MERFISH and SeqFISH (Asp *et al.*, 2020).

The method we propose has been implemented in Python and is provided as an open-source tool named *sepal* (**s**patial **e**xpression **pa**ttern **l**ocator), hosted on GitHub (https://github.com/almaan/sepal). Our implementation offers CPU parallelization, but is not designed for GPU acceleration. Means for visualization and generation of pattern families are provided by an analysis module in *sepal*.

Focusing on individual transcripts, *sepal* facilitates informative analysis of large spatial transcriptomics datasets while producing results where the biological, interpretable, components are preserved.

## 2 Materials and methods

### 2.1 Terminology

We will abandon the use of ‘gene expression profile’ in favor of the stipulative term *transcript profile*; this is to emphasize that the profiles represent a spatial arrangement of individual transcripts associated with a given gene.

### 2.2 Standard model

First, let (Ω) denote the area defined by a tissue specimen. If expression values are collected from this tissue using a structured grid, we could consider this grid as a partitioning or discretization (*S*) of the domain Ω. When referring to members (the grid points) of *S*, we use *s*. Every point *s* has a set of neighbors ($N(s,d_{P})$) defined as:

(1) $$N(s,d_{P})=\{q:q\in S,||x_{q}-x_{s}|{|}_{2}\leq d_{P}\},$$

where *d_P_* depends on the experimental platform (*P*), the platform also dictates the maximal number of members (*M_P_*) that a neighbor set may have. A grid point *s* with $|N(s,d_{P})|=M_{P}$ is called saturated, all other points are referred to as unsaturated. Together the saturated points make up the inner points (*S_i_*), the remaining set of unsaturated points are referred to as the boundary points (*S_b_*).

Next, we let the function u(x,y,t) be defined over Ω, representing the observed expression values (number of transcripts) of a certain gene (*g*) at spatial location (*x*, *y*) and time *t*.

We may then use Fick’s second law to obtain an expression for diffusion within the tissue, resulting in:

(2) $$\frac{\partial u(x,y,t)}{\partial t}=D\Delta u(x,y,t),$$

where *D* is the diffusion coefficient and Δ the Laplacian. The time point *t *=* *0 represents the initial state of the system, here the observed—and unperturbed—values. The exact form of *u* is unknown, and thus equally the value of Δu(x,y,t). Fortunately, we can approximate the Laplacian numerically. On a regular rectilinear grid this approximation is taken as (excluding the time variable for brevity):

(3) $$\begin{array}{c} \begin{array}{c} \Delta u(x,y)\approx \frac{1}{h^{2}}[u(x+h,y)+u(x-h,y)+\\ +u(x,y+h)+u(x,y-h)-4u(x,y)]. \end{array} \end{array}$$

For hexagonal grids, like those of the Visium arrays, we use a different approximation scheme presented in the work of L. Kantrovich and B. Krylov (Kantorovich, 2018). For an exact specification of the supported grids and specifications of their associated numerical methods, see Supplementary S2.1.

After the Laplacian has been approximated with Eq. 3, the system is propagated in time using the dynamics of Eq.2, effectively simulating diffusion of transcripts within the tissue, that is:

(4) u(x,y,t+dt)=u(x,y,t)+DΔu(x,y,t)dt.

Here, *dt* represents a small step in time. Values at the boundary (∂Ω) of the domain (Ω), are updated by letting the Laplacian of a boundary point be equal to that of its nearest inner point.

Furthermore, for any subset S′⊆S, we define the entropy ($H_{S\prime }$) as:

(5) $$H_{S\prime }(t)=-\sum_{s\in S\prime }\;\text{log}\;({\hat{u}}_{s}^{t})\cdot {\hat{u}}_{s}^{t},$$

 

(6) $${\hat{u}}_{s}^{t}=\frac{u(x_{s},y_{s},t)}{\sum_{k\in S\prime }u(x_{k},y_{k},t)}.$$

The system is considered converged when the average change in entropy, taken over the set of inner points (*S_i_*), between two consecutive time steps is below a certain threshold (*ϵ*). The time at which convergence occurs is referred to as the *diffusion time* (*t_d_*), hence:

(7) $$|H_{S_{i}}(t_{d})-H_{S_{i}}(t_{d}-1))|<\epsilon \times |S_{i}|.$$

The diffusion time serves as the metric by which we rank transcript profiles; high diffusion times are indicative of an organized spatial distribution. When presented, diffusion times are minmax normalized (subtraction of the smallest value and division by the range), meaning the largest value for any sample will be 1 and the smallest 0.

### 2.3 Normalization

Unless stated otherwise, expression vectors with raw values ($y_{g}$) of a given gene *g* are converted into normalized values ($u_{g}$). This occurs by first applying a log (base 2) transformation with a pseudocount *c*, to emphasize relative changes in the expression rather than absolute ones. The log transformed values are then mapped to the unit interval ([0,1]) through division with the vector’s largest element:

(8) $${\hat{y}}_{g}={\;\text{log}\;}_{2}(y_{g}+c),$$

 

(9) $$u_{g}=\frac{1}{\max\{{\hat{y}}_{g}\}}\times \hat{y_{g}}.$$

The log  and max functions are both applied elementwise. The recommended value for *c* is 2, as this is more robust toward sparse non-structured transcript profiles than 1, but the user is free to choose any positive number in our implementation. The default pseudocount is set to 2, in contrast to the common value of 1; this is to dampen the negative effects the presence of sparse transcript profiles may have on the results. Sparse profiles, having few non-zero observations, tend to require a long time to reach a homogeneous state (i.e. to converge) despite the lack of any initial non-random spatial structure, see Supplementary Figure S2.5. This is due to the artificially large gradients, that are introduced when most non-zero observations are surrounded by zero observations, an issue that becomes less prominent with a larger pseudocount.

### 2.4 Selection of top profiles

Our method assigns a rank to each transcript profile and by design do not operate with notions of significance or similar metrics. As a consequence, no dichotomization into groups of profiles with or without spatial patterns occurs. Still if such a partition is desired—e.g. for downstream analysis—we recommend the user to inspect the top-ranked profiles and set a suitable rank-cutoff (e.g. top 100) based on this. We have also implemented a simple heuristic approach that automatically will select a number of transcript profiles with distinct spatial patterns, as might be desirable in a larger analysis workflow, for more details see Supplementary S2.2. The heuristic is designed to be conservative, rather excluding profiles that exhibit some spatial structure than including those with weak or no spatial pattern.

### 2.5 Pattern families

Once a set of transcript profiles with organized patterns have been identified, we may ask which biological processes that drive these formations, i.e. what makes transcripts organize as observed. top-ranked transcript profiles could be examined individually, but to provide a more holistic representation of the results we suggest a procedure to group transcript profiles into different groups where elements display similar spatial structures. We will refer to these groups as *pattern families*. If the pattern families are subjected to functional enrichment analysis the biological processes associated with them, and indirectly the spatial structure they adhere to, may be identified. The procedure we suggest draws inspiration from the use of eigenfaces in facial recognition applications (Turk and Pentland, 1991).

First, *T* top genes (w.r.t. diffusion time) are selected, a cutoff implemented to prevent profiles with spurious spatial distributions from convoluting the analysis. We normalize expression levels within a capture location by the sum of all observations in said location (library size normalization). Next, principal component analysis (PCA) is applied to the selected top transcript profiles, from this the *k* components that explain *p* percent of the variance—computed from the eigenvalues—are used as basis vectors for a *k*-dimensional subspace.

The extracted basis vectors could be considered as *eigenpatterns*, from which the spatial transcript profiles can be assembled. In a final step, the transcript profiles are projected onto the eigenpattern subspace (spanned by the top principal components) and assorted into *k* families by agglomerative clustering, see Supplementary Section S2.3. We let the angle between the projections figure as our metric of distance. Hence, the similarity in composition of eigenpatterns is what determines the relation between expression profiles, and as a consequence, the members of each family. Representative motifs for the families are obtained by combining the eigenpatterns according to the average loadings (contributions) among the members.

### 2.6 Synthetic data

We devised two procedures to construct sets of synthetic spatial transcriptomics data, used in the assessment of our method’s performance. We refer to products from each approach as *mixed* and *ablation* sets respectively; the former being a mixture of profiles with different spatial patterns together with randomly shuffled variants of these, while the latter consist of gradually perturbed versions of a single profile.

#### 2.6.1 Mixed sets

Let P be a set of spatial expression data, where each member represents the observed expression from a given gene over |S| locations. If all members of P exhibit a spatial pattern, it’s suitable as a *seed* to generate a larger mixed set W. The mixed set is constructed by the following procedure: Each expression vector in P ($p_{i}$) will be multiplied with a multiplier (*m_j_*) to augment different expression levels (***w***) followed by a permutation (rearrangement), a procedure repeated $n_{\text{offspring}}$ times. Since element *s* of ***w*** is associated to the coordinate pair (*x_s_*, *y_s_*), the permutation effectively reorganizes the expression signals in space, what we refer to as *shuffling*. Furthermore, if $p_{i}\in P$ was used to generate $w_{k}\in W$, we denote $p_{i}$ as a parent profile and consider $w_{k}$ its ‘offspring’. Mixed sets are useful to assess a method’s ability to distinguish transcript profiles with clear spatial structure from those of more random character. Any method that identifies spatial expression patterns should rank the offsprings as less spatially structured than their parent patterns.

**Algorithm 1: Assembly of mixed set**

Let M be a set of (positive) multiples;

Let the vector $p_{i}$ represent the i:th member of P;

Let the mutltiple *m_j_* represent the j:th member of M;

Let W = ∅;

**for**  i←1  **to**  |P|  **do**  $W=W\cup \{p_{i}\}$; **for**  j←1  **to**  |M|  **do** **for**  f←1  **to**  $n_{\mathrm{offspring}}$  **do**  $w=p_{i}*m_{j}$ Randomly Shuffle ***w***;

W=W∪{w};

  **end** **end end**

#### 2.6.2 Ablation sets

In contrast to the mixed sets, which utilize multiple spatial patterns during construction, each ablation set originates from a single transcript profile. This profile is gradually perturbed by shuffling an increased number of observations, forming a sequence of expression data with an innate internal rank w.r.t spatial structure. While the mixed sets offer insights into how methods treat a collection of different spatial patterns in the presence of profiles with no structure, the ablation sets allow us to gauge whether a method can differentiate between different degrees of spatial structure. To clarify, methods devised to rank or identify spatial patterns should ideally assign higher ranks or more significant values to members of the set with a low amount of deformation (few shuffled observations).

#### 2.6.3 Seeding sets

Two different approaches were used to construct seeding sets, one image-based and the other utilizing simulation of Turing patterns. The image-based method takes a black and white image as input, where white regions represent areas of elevated expression (forming a spatial pattern). The second method generates (Turing) patterns by propagating a dynamic system in time, using random initial values, see Supplementary Section S2.4. By using these two seeding sets we aim to demonstrate our method’s performance both with profiles that we have crafted and subjectively consider as structured, as well as those produced by a stochastic process.

## 3 Results

Our method was first applied to synthetic data to confirm expected performance, followed by analysis of real data. The mode of visualization is the same for all sets of data, real and synthetic; a capture location is plotted with the expression levels indicated by facecolor. The signal values are log transformed (base 2) with pseudocount 2, consistent with the normalization applied upon analysis. See Supplementary Section S4 for analysis parameters. Colors are scaled internally within each profile, to emphasize spatial structures.

### 3.1 Synthetic data

Two mixed sets of synthetic data were constructed, $W_{1}$ and $W_{2}$. Ten hand-drawn black and white images were used to generate the seeding set ($P_{1}$) for $W_{1}$, see Supplementary Figure S3.1. For $W_{2}$, the seeding set ($P_{2}$) consisted of 10 simulated Turing patterns, see Supplementary Figure S8. For both sets, we let $n_{\text{offspring}}=3$ and M={0.5,1,2}. Thus, each mixed set consisted of 100 transcript profiles, with 10 of these exhibiting distinct spatial structure. Parameters for Turing pattern generation and images used to construct $P_{1}$ are found in Supplementary Section S3.

**Fig. 1.**
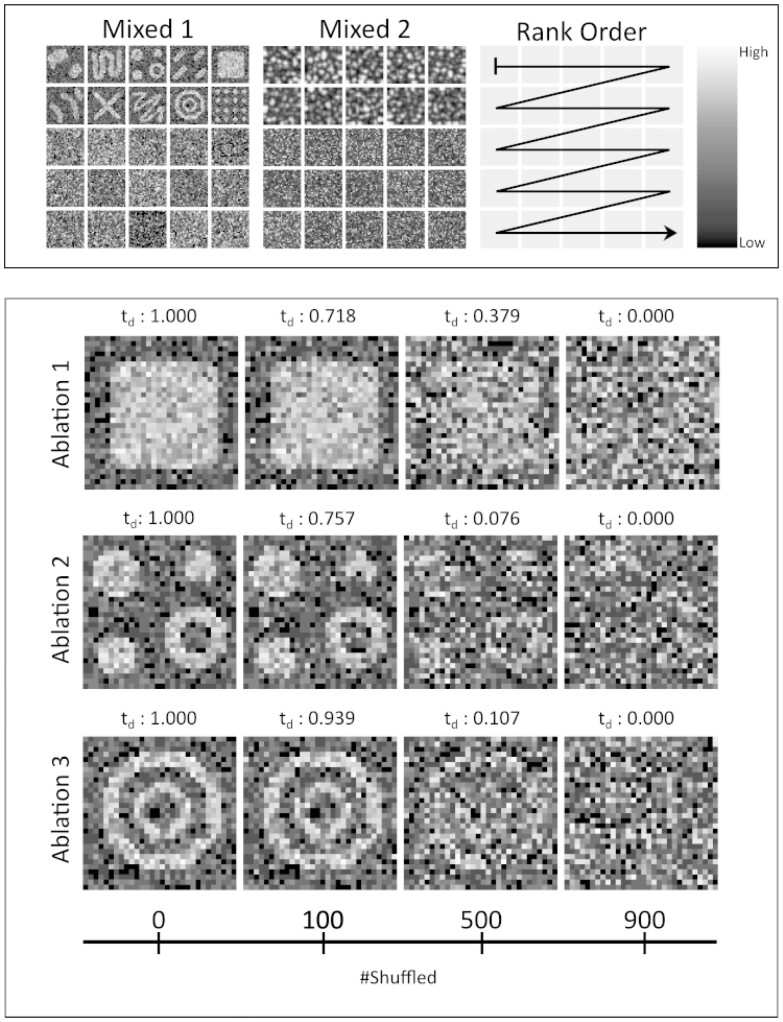
Top: The mixed sets $W_{1}$ (Mixed 1) and $W_{2}$ (Mixed 2) sorted by each member’s diffusion time (order indicated in rightmost picture). Only the top 25 synthetic transcript profiles are shown. Bottom: Ablation sets $A_{1},\;A_{2}$ and $A_{3}$ sorted by diffusion times. Normalized diffusion times are given as *t_d_*. Bottom bar indicates the number of shuffled capture locations, i.e. the extent of the perturbation

For both sets ($W_{1}$ and $W_{2}$), the 10 parent expression profiles with true spatial patterns were assigned higher rank by our method than all of the offsprings, each event having a probability of $(10!90!)/100!\approx 5.78\cdot 10^{-14}$ to occur by chance. Parts of the results are illustrated in Figure 1 where the top 25 expression profiles are given for each set, see Supplementary Figures S9 and S10 for complete results.

We also generated 10 ablation sets ($A_{1}$ to $A_{10}$), one from each of the spatial patterns in $P_{1}$. These ablation sets have 4 different degrees of perturbation—the number of shuffled observations at each stage being {0,100,500,900}. For all 10 sets, members were ranked in the correct order, an event with a probability of ${\left(4!\right)}^{-10}\approx 1.58\cdot 10^{-14}$ to occur by chance. Results from three of the ablation sets ($A_{1}$ to $A_{3}$), are presented in Figure 1. See Supplementary Figures S11 and S12 for complete results. Raw synthetic data and results are found in Supplementary Data S1.

### 3.2 Real data

We applied the method to real spatial transcriptomics data from five different types of tissue: mouse olfactory bulb or MOB (1k ST array), mouse brain (Visium), human lymph node (Visium), human melanoma (1k ST array) and mouse cerebellum (Slide-seq). Results from the Slide-seq data are available in Supplementary Section S5.8, illustrating how the method can be generalized to unstructured spatial data. Ribosomal and mitochondrial filtering was used in all analyses (see Supplementary Section S4.1), additional basic quality filtering was also applied to the expression data (see Supplementary Sections S1 and S4).

**Fig. 2.**
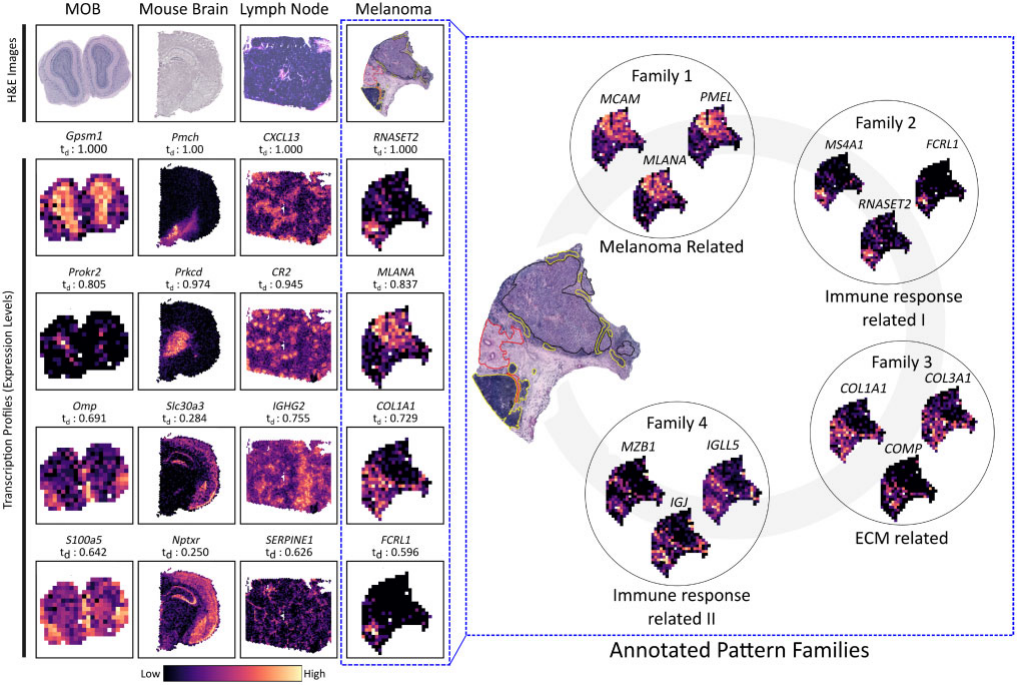
Excerpts from the set of top 20 ranked transcript profiles of each sample. Normalized diffusion times are given as *t_d_*. H&E-images (Hematoxylin and Eosine) of each sample are provided as references. transcript profiles are represented by coloring the spatial location according to the normalized expression levels; low values are black, high values are bright. Annotated pattern families for the melanoma sample (blue dashed box) are presented with three members of each family. We include the pathologist’s annotations from the original paper, where black: melanoma, red: stroma and yellow: lymphoid tissue

Figure 2 shows excerpts from the top 20 ranked transcript profiles, taken from each sample. Visualization of the top ranked profiles for all analyzed samples are available in Supplementary, complete results for all real sets are found in Supplementary Data S1. When the trancription profiles were examined along the ‘ranking-gradient’ (from top-ranked to bottom ranked) they clearly showed an increasingly random character, see Supplementary Figure S13 for an example.

We were interested in whether the top-ranked transcript profiles had any biological relevance, and therefore decided to examine some of them more thoroughly. As expected, we found that these profiles often represented marker genes of certain cell types, or genes involved in important biological pathways. To exemplify: *Prokr2* is indicative of immature interneurons that have migrated from the subventricular zone to the olfactory bulb (Puverel *et al.*, 2009; Wen *et al.*, 2019); *Omp* is a marker gene for olfactory sensory neurons (Shiao *et al.*, 2012); *Pmch* is a marker gene for a small population of neurons populating the lateral hypotalamic area of mice, observed to have influence on the animal’s behavior (Mickelsen *et al.*, 2019); *Slc30a3* encodes a zinc transporter protein (ZNT3) found in zinc-secreting neurons and, in addition to other pathways, is involved in communication between granule and pyramidal cells (Henze *et al.*, 2000; Linkous *et al.*, 2008); *CXCL13* is a chemokine targeting B-cells, and is known to be essential for the formation of lymph nodes (van de Pavert *et al.*, 2009); *CR2* encodes a receptor that partake in the complement system, and is known to be expressed by follicular dendritic cells located in lymph nodes (Kranich and Krautler, 2016).The set of top-ranked transcript profiles in the mouse brain was compared to those listed as highly variable by a common variance metric, see Supplementary Section S5.5.1. Several of the top 100 highest ranked profiles, exhibiting distinct spatial structures, were absent from the set of 1000 most variable ones, demonstrating the value of using ‘spatially-aware’ methods.

Several of the top-ranked transcript profiles in the melanoma sample appear to be associated with the disease, but were not mentioned in the original publication (Thrane *et al.*, 2018). Examples of such genes are: *RNASET2*, a known tumor antagonist in melanoma (Monti *et al.*, 2008); and *FCRL1*, encoding a glycoprotein linked to the disease’s progression (Koh *et al.*, 2012). Both these genes were abundant in regions annotated as lymphoid tissue. In comparison, transcripts of *MLANA* [an established marker of melanoma with prognostic properties (Reid *et al.*, 2013)], were more prevalent in the cancerous region.

To further assess which biological processes that could be ascribed to the patterns we observed, the 150 transcript profiles with highest rank in the melanoma sample were assorted into pattern families. A total of four pattern families were identified and subjected to functional enrichment analysis; using *g: Profiler* and querying against the GO: BP (Gene Ontology, Biological Processes) database (Raudvere *et al.*, 2019). The complete list of enriched processes can be found in Supplementary S5.7 together with visualization of the four families and their representative motifs. Family 1 was enriched for multiple processes related to cell growth and differentiation; this in conjunction with multiple genes in the family being directly associated to melanoma led us to annotate it as ‘melanoma related’. This is also in concordance with the annotations provided by the pathologist (given in the original publication). Family 2 and 4 were enriched for immune response related pathways; the former had more general processes of cell activation (lymphocyte and leukocyte) and regulation associated with it, while specific immunoglobulin related processes were listed for the latter; we annotated both these as immune response related families (with identifiers I and II respectively). Family 3 was enriched for collagen constituents, processes involved in collagen organization, and platlet activation, hence its annotation as ‘ECM (Extracellular Matrix) related’. Evidently, not every biological process present in the tissue sample can be expected to have an associated spatial pattern, but this approach illustrates how certain functionality can be linked to spatial patterns we observe in the data.

The landscape of methods to find expression profiles with spatial patterns may be sparsely populated, but alternative methods to ours do exists. Two examples of such methods are SpatialDE and SPARK (Sun *et al.*, 2020; Svensson *et al.*, 2018); which both rely on hypothesis testing to produce sets of genes with statistically significant spatial patterns. Thus, we were interested in how our approach compared to these alternative and fundamentally different methods. The MOB sample included in our study, was also examined in the publication of SpatialDE as well as that of SPARK, hence it was a natural choice for the comparison. The result from applying SpatialDE to this particular sample was downloaded from its associated GitHub repository. Similarly for SPARK, code to reproduce the MOB analysis was available at its repository; we executed these scripts without any modifications (see Supplementary S4.4). For the alternative methods we used statistical significance as a rank metric; the more significant, the higher the rank. The top 20 ranked expression profiles from each method were inspected (see Supplementary Figs S26 and S27), by doing so we noted that all methods successfully presented transcript profiles with organized spatial patterns. However, the top profiles of SpatialDE and SPARK both included genes that were relatively homogeneously expressed over the tissue (e.g. *Apoe, Sparcl1* and *Glul*), not present among the top transcript profiles of our method. Next, aware of how genes with high expression levels tend to overlap with those exhibiting structured spatial arrangement, we asked how prevalent this phenomena was in the result from respective method. This is of relevance because; if expression levels is what informs the ranking, the identified expression profiles could just as well be obtained from sorting the genes w.r.t to expression levels. We addressed this question by computing the Spearman (*ρ*) correlation between the metric by which profiles were ranked and the total observed counts of the genes, see Supplementary Section S4.4. The magnitude (absolute value) of the correlation was lowest in our method (|ρ|=0.1399), about half that of SPARK (|ρ|=0.2576) and somewhat lower than SpatialDE (|ρ|=0.1877), see Supplementary Table S4. This suggests that our method is less dependent on expression levels when ranking transcript profiles compared to alternative methods.

To better understand what qualitatively differentiates our method from the others, we examined transcript profiles uniquely identified in respective method as well as those listed by both SPARK and SpatialDE but not ours, see Supplementary Figs. S29–S32. What can be discerned from this analysis is how our method favors transcript profiles with a pronounced contrast between pattern and background, an immediate consequence of its design. This allows it to detect irregular and ‘thin’ patterns that the other methods might overlook (e.g. *Sox11*), but also makes it less appropriate for cases where small discrepancies between pattern and background is expected (e.g. *Calm2* and *Synpr*).

We also compared performance (with respect to runtime) of the three aforementioned methods using 1–4 CPU cores. When analyzing the MOB sample, our method was faster than both SpatialDE and SPARK in all instances except one; being when a single core was used, in this case SpatialDE completed the analysis faster than *sepal*, for more details see Supplementary Section S5.10.

## 4 Discussion

We have developed a theoretical model for unsupervised identification of transcript profiles that exhibit spatial patterns, in response to the emerging need to separate relevant spatial signals from noise. The model is implemented in Python and released as an open-source tool, *sepal*, with support for data originating from multiple platforms and modules for additional analysis. We base the method on numerical simulation of transcripts diffusing within the tissue, using Fick’s second law. Like any method that operates with patterns of individual transcript profiles, ours do not require interpretation of abstract entities such as clusters or factors. Still, the diffusion-based approach we present stands in contrast to previous methods, where characterization of the spatial distribution and hypothesis testing tend to figure as core concepts. In short, we present a novel theoretical framework with high interpretability that enables unsupervised exploratory examination of large sets of data. The method performed well on multiple synthetic and real datasets. Spatial expression patterns were ranked higher than all their related random patterns (as desired), both when generated from hand-drawn images as well as simulated Turing patterns. Members of the 10 ablation sets—where we perform a gradual deformation of structured spatial transcript profiles—were also ranked in the expected order. Analysis of real data from different tissue types and techniques, resulted in a set of transcript profiles with clear spatial patterns and biological relevance. Our method is an important complement to techniques where analysis relies on more abstract or coarse entities such as factors and clusters, since attention is brought to genes with structured spatial arrangements which otherwise might be overlooked. When compared to other existing methods designed for the same purpose, ours were equally capable of finding expression profiles with spatial patterns, but seems to be less driven by the extent to which a gene is expressed and rather by its spatial organization. A procedure to group genes with similar spatial expression profiles into pattern families is also suggested and included as an analysis module in our implementation. Aggregating profiles into pattern families and subjecting them to enrichment analysis may lead to functional annotation of spatial regions, as illustrated by our study of the melanoma sample, and understanding of how certain biological pathways propagate through the tissue. Previous methods have also grouped genes by clustering them based on expression levels, however these do not make use of the intermediary space of eigenpatterns that we propose. Since *sepal* operates by ranking profiles, the presence of a few dominant patterns may quench other, less strong, but still relevant patterns. We see such tendencies in the mouse brain sample, where several of the top transcript profiles share a similar structure. Sorting the expression profiles into patterns families can to some extent mitigate this issue, but it is an inherent feature of the method’s design that should be acknowledged. While the method has been developed for and demonstrated with gene expression data, it could in theory be generalized to any type of data where measurements or inferred values are associated with given spatial positions; one relevant example of this being cell type identities. We consider the initial exploratory phases spatial transcriptomics studies as those where *sepal* can bring most value; guiding the user toward genes worth pursuing for further analysis. *sepal* is available as a Python package at GitHub (https://github.com/almaan/sepal), together with documentation, tutorials and all scripts used to produce the material in this paper.

*Financial Support*: none declared.

*Conflict of Interest*: none declared.

## Supplementary Material

btab164_Supplementary_Data
