# Supplementary material for: sepal: identifying transcript profiles with spatial patterns by diffusion-based modeling
Source: Bioinformatics. 2021 Mar 11;37(17):2644–50. doi: 10.1093/bioinformatics/btab164 (PMC8428601; doi:10.1093/bioinformatics/btab164)
Supplement: btab164_Supplementary_Data [file btab164_supplementary_data.pdf]

sepal : Identifying Transcript Profiles with Spatial Patterns by Diffusion-based Modeling

Alma Andersson, Joakim Lundeberg

2021-03-07

Contents

S1 Data **2**

S2 Methods **2**
S2.1 Supported Data . . . 2
S2.1.1 Hexagonal Arrays . . . 2
S2.1.2 Unstructured Data . . . 3
S2.2 Selection of Top Profiles . . . 3
S2.2.1 Sensitivity Parameter . . . 3
S2.2.2 Evaluation . . . 5
S2.3 Hierarchical Clustering . . . 6
S2.4 Synthetic Data . . . 6
S2.4.1 Image Based . . . 6
S2.4.2 Turing Patterns . . . 6
S2.5 Pseudocount Choice . . . 7

S3 Seeding Sets **9**
S3.1 Seeding Set 1 . . . 9
S3.2 Seeding Set 2 . . . 9

S4 Analysis **9**
S4.1 Filtering . . . 9
S4.2 Diffusion Model . . . 10
S4.3 Melanoma : Pattern Families . . . 10
S4.4 Comparison . . . 10

S5 Results **11**
S5.1 Mixed Set 1 . . . 11
S5.2 Mixed Set 2 . . . 12
S5.3 Ablation Sets . . . 13
S5.4 MOB . . . 15
S5.5 Mouse Brain . . . 17
S5.5.1 Comparison with highly variable genes . . . 17
S5.6 Lymph Node . . . 19
S5.7 Melanoma . . . 20
S5.7.1 Top transcription Profiles . . . 20
S5.7.2 Representative motifs . . . 21
S5.7.3 Pattern Families . . . 22
S5.8 Mouse Cerebellum . . . 26
S5.9 Comparison . . . 27
S5.10Performance Benchmarking . . . 34

S1 Data

The five public data sets used in our study were accessed via the following links:

- MOB : `https://www.spatialresearch.org/resources-published-datasets/doi-10-1126science-aaf2403/` (Rep11)
- Mouse Brain : `https://support.10xgenomics.com/spatial-gene-expression/datasets/1.0.0/V1_Adult_Mouse_Brain`
- Lymph Node : `https://support.10xgenomics.com/spatial-gene-expression/datasets/1.0.0/V1_Human_Lymph_Node`
- Melanoma : `https://www.spatialresearch.org/resources-published-datasets/doi-10-1158-0008-5472-can-18-0` (ST_mel1_rep1)
- Cerebellum : `https://singlecell.broadinstitute.org/single_cell/data/public/SCP354/slide-seq-study` (Cerebellum_Puck_180819_11)

The synthetic data sets used to assess the performance of our method are found at the github repository within the folder "synthetic-data" as well as in Supplementary Data 1.

S2 Methods

We here elaborate more on the types of supported data and how numerical approximations are performed, the selection of top transcription profiles, the hierarchical clustering and the generation of synthetic expression profiles.

S2.1 Supported Data

We have implemented support for three different types of data:

1. Capture with rectangular arrays
2. Capture with hexagonal arrays
3. Unstructured capture

The original ST arrays utilize (1), the Visium platform from 10x^{TM} relies on (2) and methods like Slide-seq (3). We have already described how the numerical approximations are obtained for (1) in the main text and to avoid redundancy we will here therefore only elaborate on (2) and (3).

S2.1.1 Hexagonal Arrays

We refer to grids where each node have six equidistant first order neighbours arranged with $\pi/3$ radians apart, as simply *hexagonal* grids. See figure 1 for a visual representation of such grids.

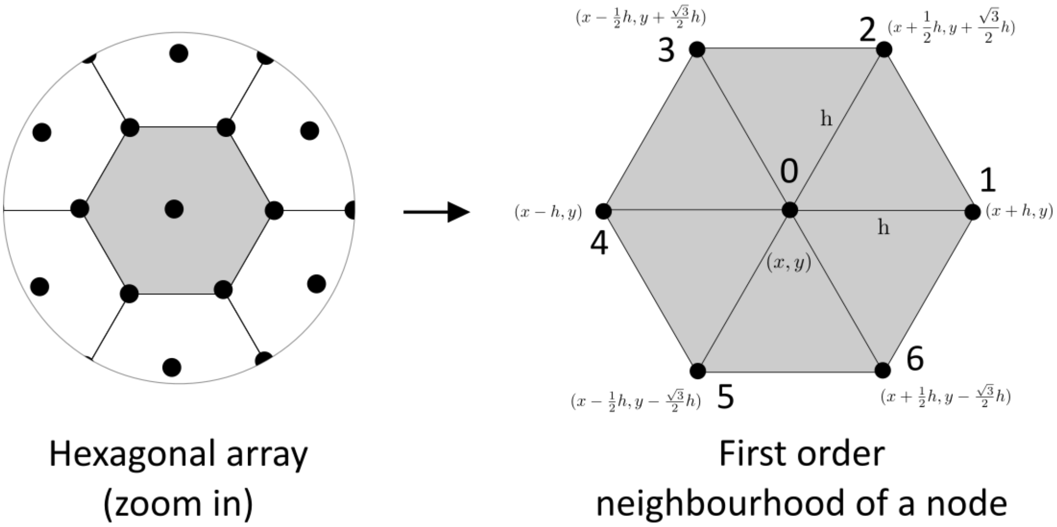

Supplementary Figure 1: Example of hexagonal array, numbers indicate node identity, and tuples coordinates.

With u being a function defined over the grid, we will let u_i denote the value of u at the $i:th$ node, for example $u_0 = u(x,y)$. From the work of Krylov and Kantrovich, we have that:

$$\sum_{i=1}^{6}(u_i - u_0) = \sum_{i=1}^{6} u_i - 6u_0 = \frac{3h^2}{2}\Delta u + \frac{9}{16}\Delta\Delta u + \mathcal{O}(h^6) \tag{1}$$

Hence,

$$\frac{2}{3h^2}\Big[\sum_{i=1}^{6} u_i - 6u_0\Big] \tag{2}$$

Serves as an approximation of the laplacian for these hexagonal grids, with an error in the magnitude of h^2, we use this seven point stencil in our implementation.

S2.1.2 Unstructured Data

In some techniques (e.g., Slide-seq), capture locations are not arranged in a structured grid, but found at arbitrary locations which varies between experiments. Even though this design is not optimal for the type of analysis we perform, we propose a method to transform and cast it into a compatible format.

We again consider the area covered by our tissue specimen (Ω) as a domain in $\mathbb{R}^2$, but where the discretization is not determined by the capture locations. In contrast to the procedure described above, for a data set with N capture locations we first construct a regular grid (S) with $M = \lceil\sqrt{N}\rceil^2$ grid points and then map each capture location to one of these. In order to create a injective map between grid points and capture locations we formulate this as a linear programming (LP) problem formulated as :

$$(P)\begin{cases} \text{min} & \sum_i \sum_j c_{ij} x_{ij} \\ \text{subj. to} & \sum_j x_{ij} = 1, \quad x_{ij} \in \{0,1\} \end{cases} \tag{3}$$

Here, $X \in \mathbb{R}^{NxM}$ is an assignment matrix where $x_{ij} = 1$ indicates that capture location i has been assigned to grid point j. C is a cost matrix, meaning that c_{ij} represents the cost of moving capture location i to grid point j. If $\boldsymbol{r}_i$ represents the i:th capture location's coordinates and $\boldsymbol{s}_j$ the j:th grid point, we define the cost matrix as:

$$C = [c_{ij}], \qquad c_{ij} = ||\boldsymbol{r}_i - \boldsymbol{s}_j||_{L_2} \tag{4}$$

Solving this problem (P) results in a defined injective map between capture location to grid points. Subsequently we "move" each capture location according to this map. Having transformed the unstructured data to a structured collection of points, we can apply the diffusion approach described above for data captured using a rectangular grid.

S2.2 Selection of Top Profiles

As described in the main text, we devise a heuristic to automatically select a set number of transcript profiles with distinct spatial patterns. This is done by identifying the "elbow point" in the curve formed when treating diffusion values as a function of their rank. For this purpose we use the "Kneedle algorithm", implemented in the python package *kneed* (https://githubcom/arvkevi/kneed). The elbow point is taken as a threshold value, meaning that profiles with a rank higher than this point will be considered as having a strong spatial pattern; we aim to make this procedure fairly conservative, meaning that exclusion of less pronounced spatial patterns is to prefer over inclusion of profiles with low or no structure.

More specifically we use the *KneeLocator* function from *kneed*, with the parameter values : *curve = "convex"* and *direction = "decreasing"*. As for the *sensitivity* parameter – which determines the stringency of the algorithm – we use a default value of 1.5; for more details regarding this choice and its effect on the number of top profiles, see Supplementary Section S2.2.1.

S2.2.1 Sensitivity Parameter

The sensitivity (hereafter σ) has an impact on the number of selected top profiles; the larger the value the more profiles will be chosen (as the algorithm becomes more conservative when calling elbow points). In order to choose a default value, we had two criteria for σ when applied to real data: (1) small perturbations of σ should only render small or no effects on the number of top profiles (it's a stable point), and (2) the profiles obtained from using σ should be members of the top-set where

distinct differences among the profiles' diffusion times can be observed. To elaborate some on (2), this would constitute the profiles in the "upper arm" and "bend" of the elbow-curve, see red region of Supplementary Figure 2.

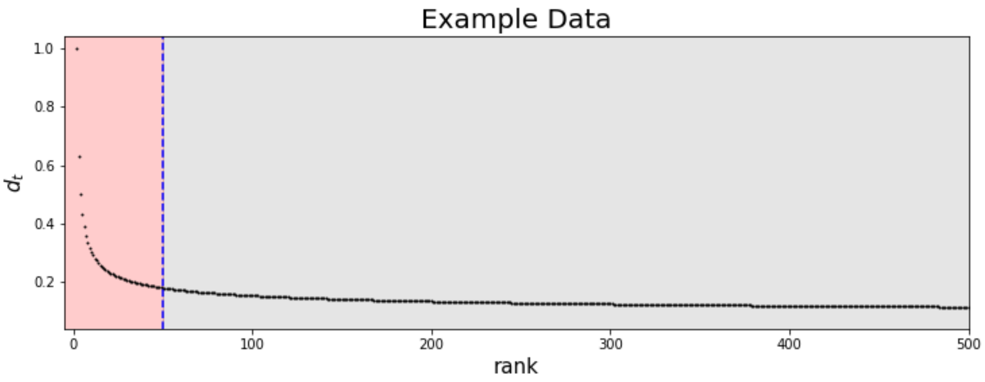

Supplementary Figure 2: Illustration of the region (red) to which the top selected genes preferably should fall within when using the rank threshold value (blue) obtained from a given σ value.

We examined how the number of top selected profiles varied depending on the σ value, and noted that for values near $\sigma = 1.5$, this number remained fairly constant – thus satisfying the first criteria. Supplementary Figure 3 shows these results, where it is evident that $\sigma = 1.5$ resides within a plateau of the curve.

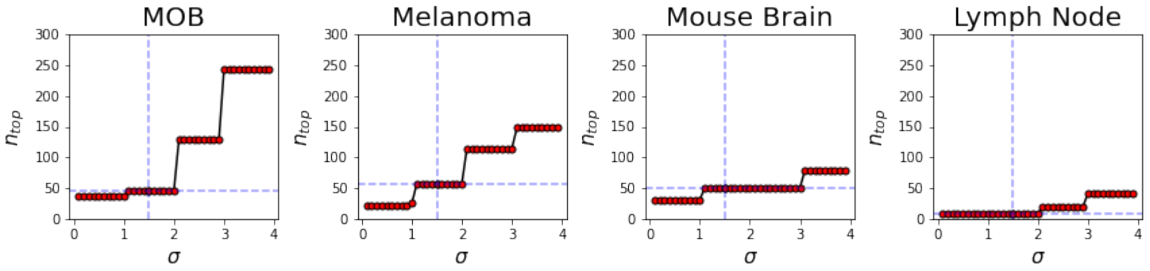

Supplementary Figure 3: Number of top selected genes as a function of the bandwidth value (σ). The σ values are separated by 0.1 units. Guides to indicate values for $\sigma = 1.5$ are included as blue dashed lines.

Furthermore, we could see how $\sigma = 1.5$ led to a selection of top profiles that made up a subset of the region specified to satisfy the second criterion (2). The results which we base this statement on are – for the four real structured data sets – shown in Supplementary Figure 4.

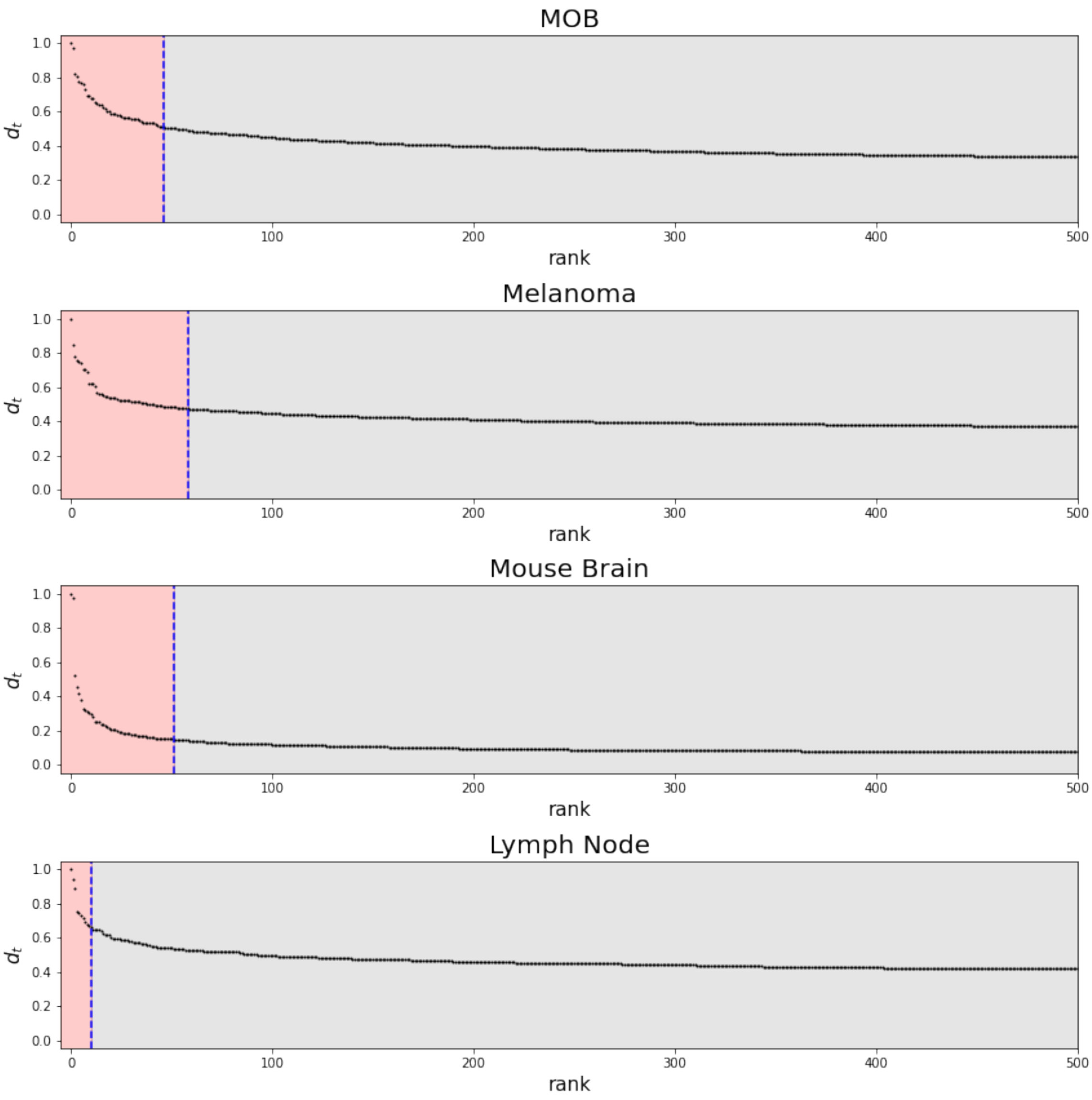

Supplementary Figure 4: Rank plots for all real data sets. The threshold rank value, with $\sigma = 1.5$, at which the partition of the profiles occur is indicated by a blue dashed line, the region where all top selected profiles reside is colored by red.

Based on these observations we deemed 1.5 to be a reasonable default value for σ, but do not claim it to be optimal for all types of data.

S2.2.2 Evaluation

We used the sets $\mathcal{W}_1$ (Image Based) and $\mathcal{W}_2$ (Turing Based) to evaluate the performance of the heuristic procedure with the default value derived from the real data. Upon applying the thresholding cutoff to the synthetic data sets, the set of profiles with "strong spatial patterns", i.e., above the threshold, in both cases solely consisted of the 10 expression profiles with true spatial patterns; that is $FPR = 0.0$ (False Positive Rate), $FNR = 0.0$ (False Negative Rate), $TPR = 1.0$ (True Positive Rate) and $TNR = 1.0$ (True Negative Rate). This is illustrated in Supplementary Figure 5.

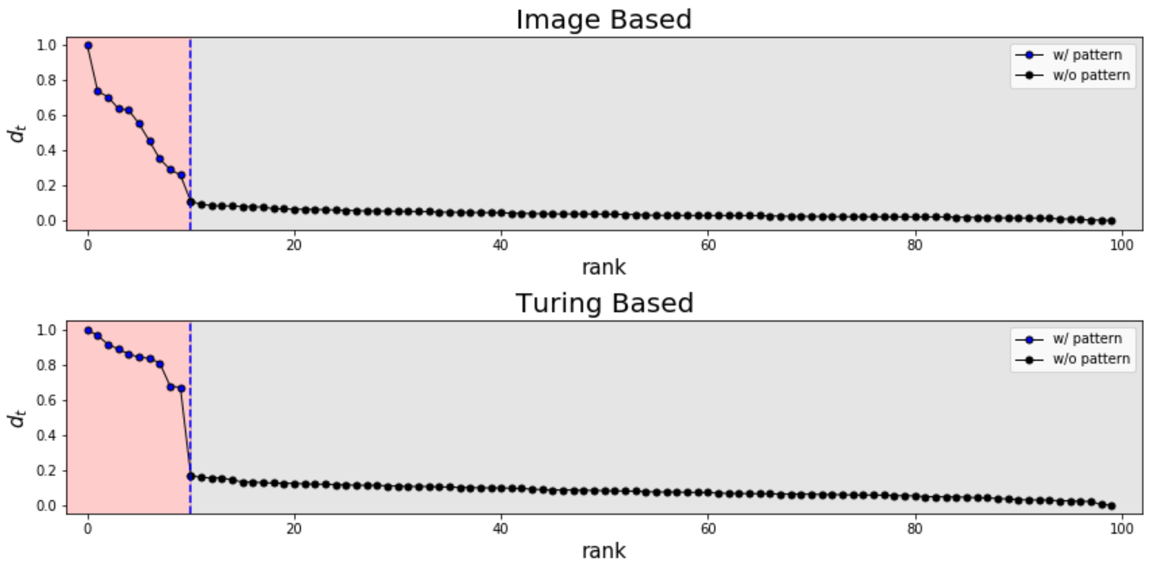

Supplementary Figure 5: Rank plots for $\mathcal{W}_1$ (Image Based) and $\mathcal{W}_2$ (Turing Based). The threshold rank value, with $\sigma = 1.5$, at which the partition of the profiles occur is indicated by a blue dashed line, the region where all top selected profiles reside is colored by red. Markers with blue facecolor indicate profiles with true spatial patterns, black facecolor with random spatial organization.

S2.3 Hierarchical Clustering

To cluster the projections, we used the *AgglomerativeClustering* class provided by sklearn (version 0.22.1). The number of clusters (*n_clusters*) was equal that of the number of identified eigenpatterns. Rather than using any of the predefined distance metrics we used the angle between each pair of projections, meaning that the *affinity* parameter was set to "precomupted". Complete linkage was used (i.e., *linkage* parameter set to "complete"). All other parameters were set to default values.

S2.4 Synthetic Data

Two approaches to generate synthetic expression data with spatial patterns have been devised, allowing us to generate what we refer to as seeding sets: an image respectively stochastic Turing pattern based approach. Here we describe these two process in more detail.

S2.4.1 Image Based

Given that I is a $n \times n$ px sized black and white image, we let each pixel represent a grid point in a structured grid. With intensity values of I residing between $[0, 255]$ we threshold the image accordingly:

$$I_{thrs} = \lfloor I/127 \rceil * 255 \tag{5}$$

Where all arithmetic operators are applied elementwise. The white areas are indicate elevated expression (forming a pattern) while black regions serve as a background. n_{pat} indicates the number of pixels (or grid points) belonging to the pattern region, and n_{bg} the same but for the background. We also define an average expression value for the pattern (μ_{pat}) and background (μ_{bg}). Finally, we randomly distribute $\mu_{pat} n_{pat}$ observations to the grid points included in the pattern region, and $\mu_{bg} n_{bg}$ over the background region. The pixel locations are used as the array coordinates and the assigned value to each of can be interpreted as expression values.

S2.4.2 Turing Patterns

We define a $n \times n$ grid, and assign a value $u^0(x_i, y_i) \sim \mathcal{U}(0, 1)$ to the $i : th$ grid point with coordinates (x_i, y_i). The exact same procedure is used to generate a second matrix V^0 with a value paired to each grid point. We consider $u^0(x, y)$ and $v^0(x, y)$ as our initial values for in a system with the following dynamics :

$$\begin{aligned} \frac{\partial u}{\partial t} &= u(1-u) - \tfrac{uv}{u+\alpha} + D_u \cdot \Delta u \\ \frac{\partial v}{\partial t} &= v\delta(1 - \tfrac{\beta v}{u}) + D_v \cdot \Delta v \end{aligned} \tag{6}$$

Where we propagate the system in time in a fashion similar to that of the diffusion model:

$$\begin{aligned} u^t &= u^{t-1} + dt \cdot \frac{\partial u}{\partial t}\bigg|_{t-1} \\ v^t &= v^{t-1} + dt \cdot \frac{\partial v}{\partial t}\bigg|_{t-1} \end{aligned} \tag{7}$$

We apply Von Neumann boundary conditions, i.e., for $f \in \{u(x,y), v(x,y)\}$ with $(x,y) \in [0,n] \times [0,n]$:

$$\begin{aligned} \frac{\partial f(0,y)}{\partial t} = 0 \quad \frac{\partial f(n,y)}{\partial t} = 0 \quad \forall y \\ \frac{\partial f(x,0)}{\partial t} = 0 \quad \frac{\partial f(x,n)}{\partial t} = 0 \quad \forall x \end{aligned} \tag{8}$$

Having propagated the system for n_{steps} number of times, the values at each grid point are multiplied by 100 and taken as expression values.

S2.5 Pseudocount Choice

As described in the main text (Section Normalization), we apply a log-transformation to our data where a pseudocount (c) is used. This pseudocount is free for the user to choose, even though we recommend a value larger than 1. This is to dampen the effects of sparse transcript profiles, i.e., profiles with few non-zero observations. Sparse genes will introduce artificially large expression gradients, since the few non-zero observations usually are surrounded by zero-values observations. As a consequence of this, they will take longer time to converge and therefore be given a high rank despite not having an initial structure of "pattern-like" character, as shown in Supplementary Figure S2.5.

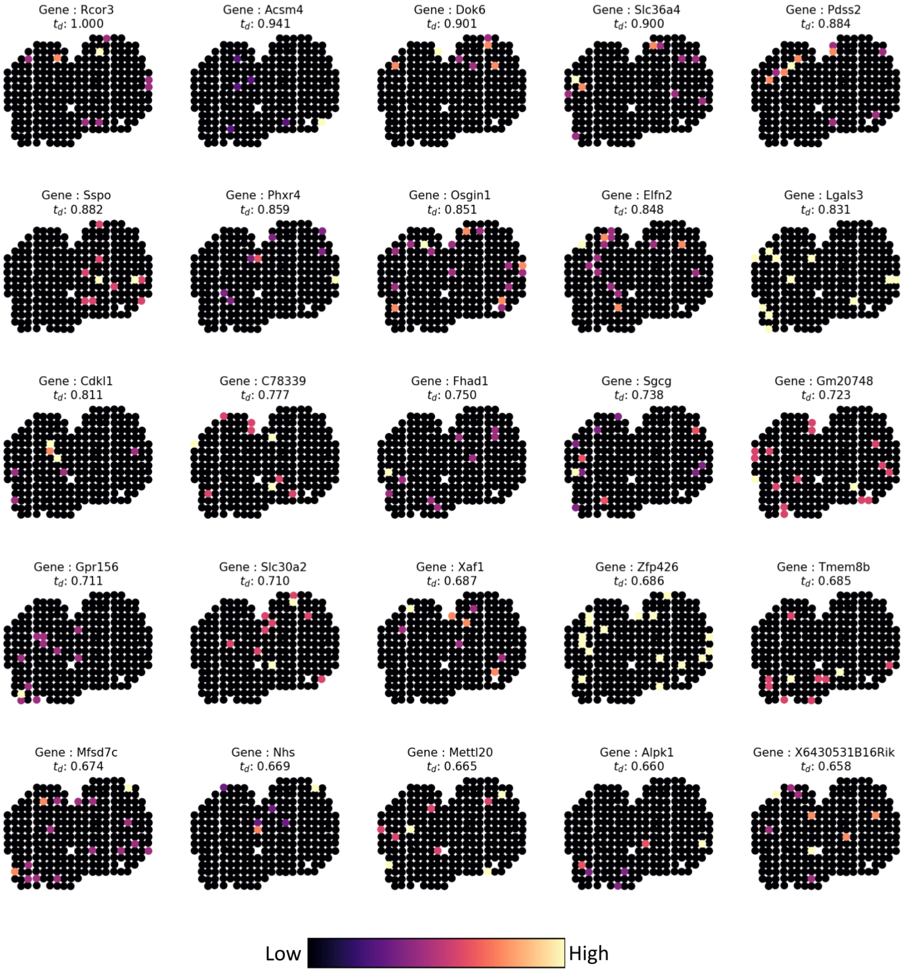

Supplementary Figure 6: Top 25 highest ranked transcript profiles (by *sepal*) for the MOB sample when using pseudocount $c = 1$ with all other parameters equal to those given in Supplementary Table 2.

sepal also supports filtering of sparse genes, where genes with more than a specified percentage of observations being zero are removed. This filtering criterion can be beneficial to implement if sparse profiles occur among the top-ranked profiles; it is also motivated by the fact that very sparse data is poorly approximated by a smooth function and hence the stencil-based approximations of the Laplacian are likely inaccurate.

S3 Seeding Sets

This section describes the exact settings used to generate the synthetic data sets used in our assessment of the method, based on the two procedures described in section S2.4.

S3.1 Seeding Set 1

The 10 hand drawn black and white images used to generate $\mathcal{P}_1$ (seeding set 1) are given in S3.1 together with the synthetic transcription profiles constituting the seeding set. We set the average expression level for the background (μ_{bg}) to 2 and that for the pattern (μ_{pat}) to 8.

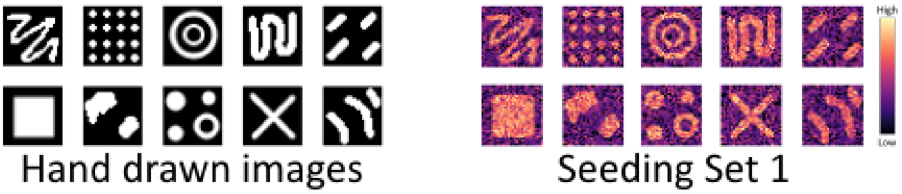

Supplementary Figure 7: **Left** : Hand drawn, black and white, images used to generate synthetic spatial expression profiles according to the procedure described in Supplementary Section S2.4.1. White regions indicate elevated expression while dark regions represent the background. **Right** Visualization of the resulting transcript profiles constituting $\mathcal{P}_1$ (Image Based seeding set), generated from the images shown in the left panel.

S3.2 Seeding Set 2

To generate $\mathcal{W}_2$ (seeding set 2) we used the approach described in S2.4.2. The parameter values used when defining the dynamical system given in Eq. 6 are given in Table 1. The system was propagated

α	D_u	D_v	β	δ
0.1	0.1	7	0.25	0.2

Supplementary Table 1: Parameter values used to generate $\mathcal{P}_2$

in time using 1000 steps and with a stepsize (dt) of 0.01. The 10 generated Turing patterns are visualized in Supplementary Figure 8.

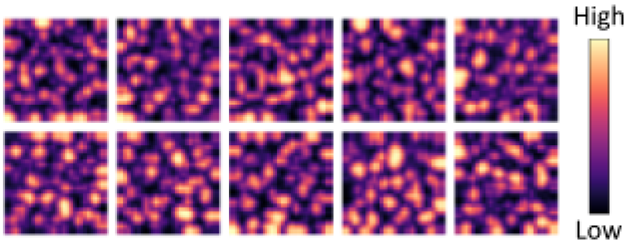

Supplementary Figure 8: Visualization of the transcription profiles constituting $\mathcal{P}_2$ (Turing Based seeding set).

S4 Analysis

This section provides information regarding the settings used for the model and subsequent analysis for the results presented within the paper.

S4.1 Filtering

Three different types of filtering were applied throughout the analysis of the samples:

- **Min. Occurrence** : minimal number of distinct capture locations that a transcript must be observed at in order to be included in the analysis.

- **Min. Total Expression** : minimal total expression values (summed over all capture locations) for a transcript to be included in the analysis
- **Max. Zero Percentage** : The maximal percentage (of the total amount of spots) allowed to have zero values for a given gene. The default value is 1.0 but for data where very sparse genes might be present or if a pseudocount of 1 is used, we recommend to adjust this value slightly.
- **RP/MT filtering** : Certain ribosomal and mitochondrial transcripts are removed by matching the uppercase name against the regular expression "ˆRP|ˆMT" (only effective if gene symbols are used as names).

Sample	Min. Occurrence	Min. Total Expression	Max. Zero percentage	RP/MT filtering	dt	c (pseudocount)
$\mathcal{W}_1$ **and** $\mathcal{W}_2$	1	1	1	No	0.001	2
$\mathcal{A}_1$ **to** $\mathcal{A}_{10}$	1	1	1	No	0.001	2
**MOB**	5	10	1	Yes	0.001	2
**Mouse Brain**	10	1	1	Yes	0.01	2
**Lymph Node**	10	1	1	Yes	0.01	2
**Melanoma**	1	1	1	Yes	0.001	2
**Cerebellum**	10	10	1	Yes	0.001	2

Supplementary Table 2: Filtering settings for each sample/set analyzed in this work.

S4.2 Diffusion Model

For the remaining parameters of the diffusion model, we used the same settings for all analyzed data sets (real as well as synthetic), with the threshold for convergence (ϵ) set to 10^{-8} and 10^{-9} for real respectively synthetic data, while the diffusion rate (D) was taken as 1.

S4.3 Melanoma : Pattern Families

Upon extracting pattern families for the melanoma sample we used the top 150 genes ($T = 150$) w.r.t to their diffusion time and required that the eigenpatterns should explain 85% of the observed variance (i.e., $p = 0.85$).

S4.4 Comparison

To compare *sepal* with SpatialDE and SPARK we followed the examples provided at the GitHub repository of respective method:

1. SpatialDE : `github.com/Teichlab/SpatialDE`
2. SPARK : `github.com/xzhoulab/SPARK-Analysis`

For the SpatialDE sample, we used the results presented by its authors in the GitHub repository (MOB_final_results.csv). As for SPARK we used the processed data they provided in their repository together with the code they present for analysis of this specific sample. The code for comparison is found in *sepal*'s GitHub repository.

The genes included in the subsequent analysis were those found in the intersection of all genes for which a rank had been assigned to in respective method. The metric used to rank the transcription profiles were: diffusion time (*sepal*), q-value (SpatialDE) and combined/adjusted p-value (SPARK). Next, for each method we computed the Spearman correlation between a gene's total observed gene expression (over all spots) and the value of its rank metric. This was done using the *spearmanr* function from scipy's *stats* module, all parameters set to default.

High values of the diffusion time results in a higher rank by *sepal*, for SpatialDE and SPARK low

q/p-values have the same interpretation. This means that for *sepal* a strong positive Spearman correlation would be indicative of rank being driven by expression levels; in contrast, a strong negative Spearman correlation would be expected for the other two methods. We therefore speak in terms magnitude when comparing the correlation values.

S5 Results

Here more elaborate information regarding the results from our analysis of the real and synthetic data is found. We first present, the results from analyzing the two mixed synthetic data sets, and the ablation sets. For the real data we visualize the top 20 transcription profiles as ranked by *sepal* for each analyzed real sample. In addition to this we have included : a visualization of the expression profiles along the ranking gradient for the MOB sample, in order to show how these compare between high, middle and low ranked profiles; the four pattern families of the melanoma sample and identified enriched processes associated to these; and a comparison between *sepal*, SpatialDE and SPARK.

The results in their raw format, i.e., the output from *sepal*, can be accessible at `https://github.com/almaan/sepal/res`.

S5.1 Mixed Set 1

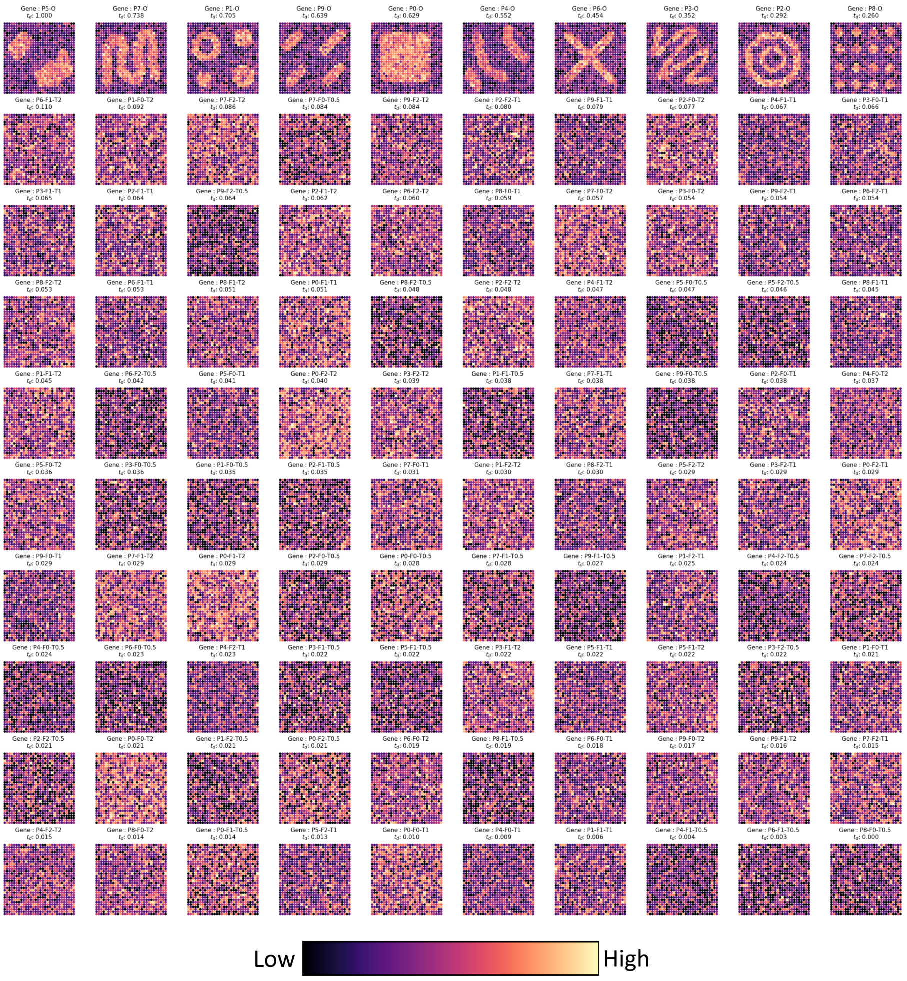

Supplementary Figure 9: Members of $\mathcal{W}_1$ ranked by their diffusion time. For each transcript profile, the header gives the diffusion time (t_d) and the "name" (Gene). Gene names are constructed as follows: P : Seeding pattern which the offspring is derived from, T : multiple of original expression level, F : permutation number (for each multiple).

S5.2 Mixed Set 2

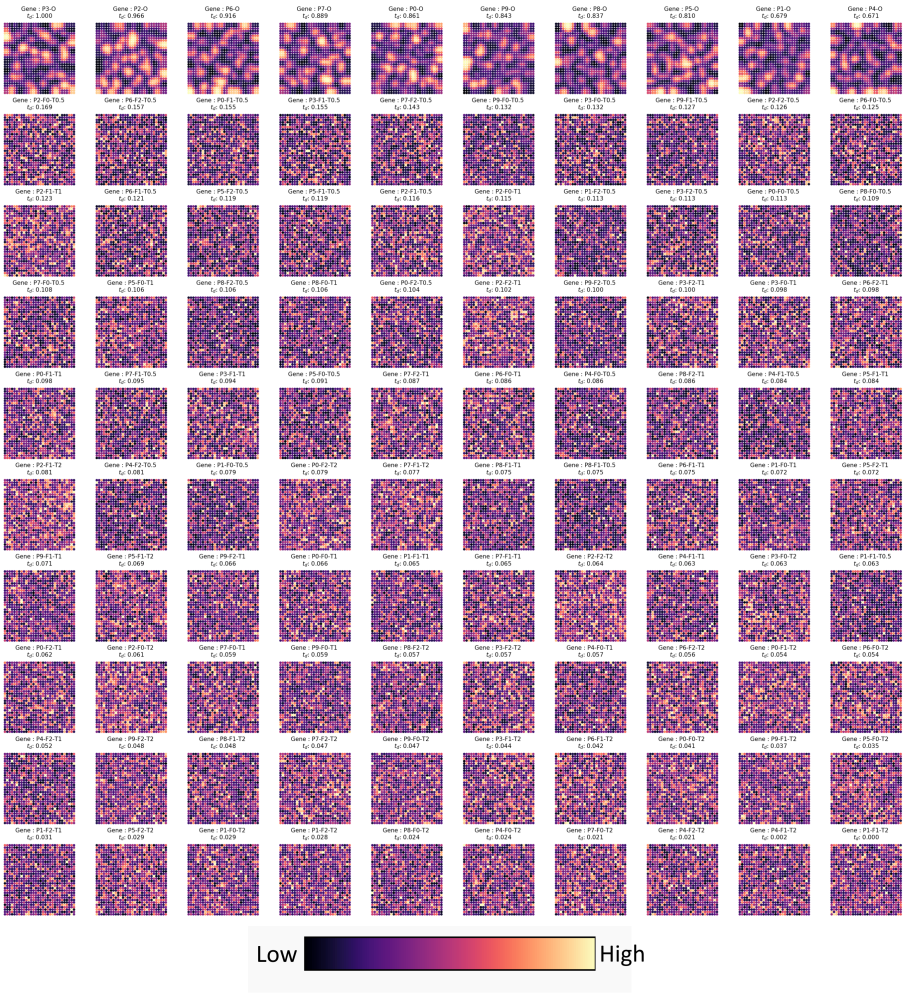

Supplementary Figure 10: Members of $\mathcal{W}_2$ ranked by their diffusion time. Members of $\mathcal{W}_1$ ranked by their diffusion time. For each transcript profile, the header gives the diffusion time (t_d) and the "name" (Gene). Gene names are constructed as follows: P : Seeding pattern which the offspring is derived from, T : multiple of original expression level, F : permutation number (for each multiple).

S5.3 Ablation Sets

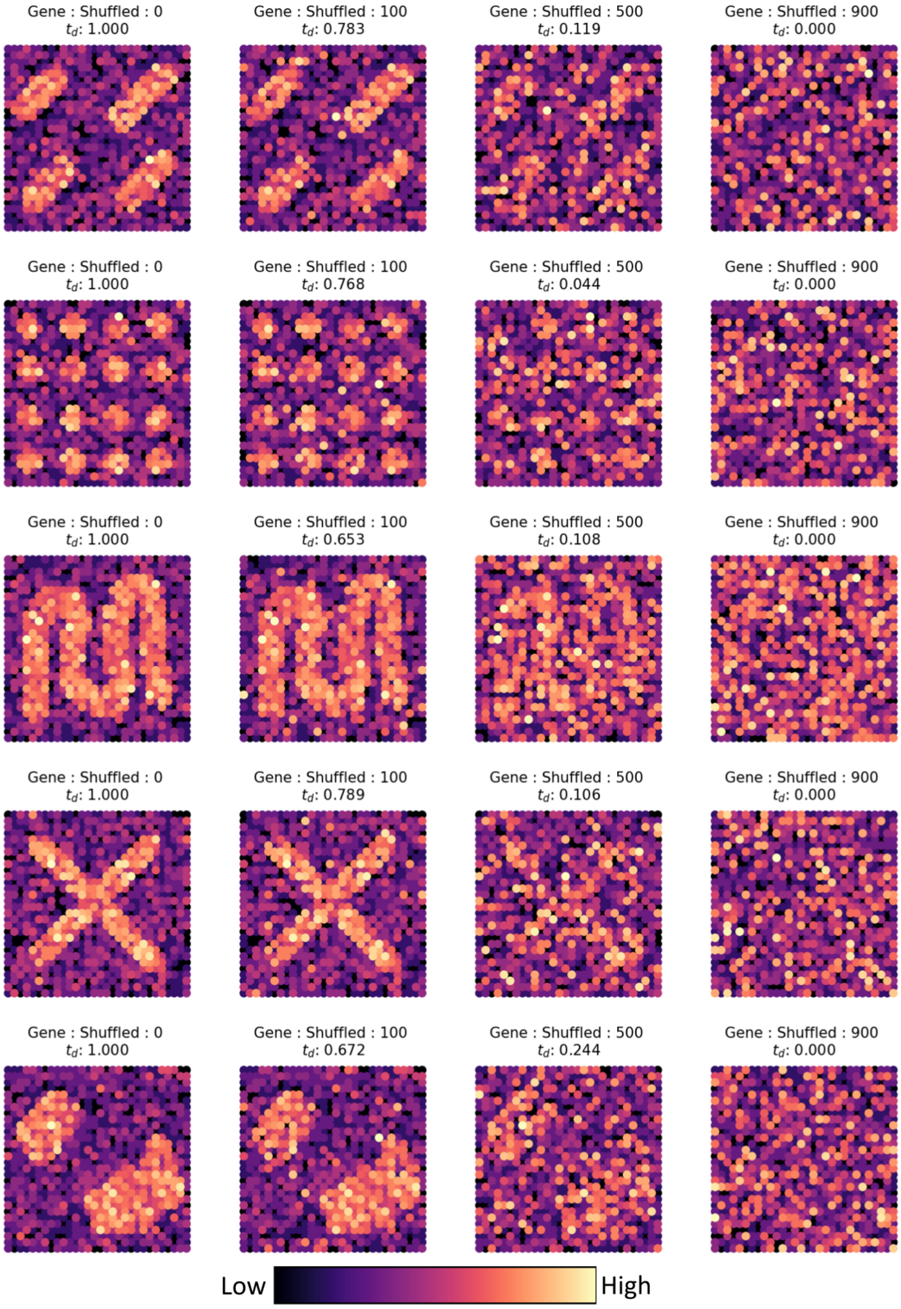

Supplementary Figure 11: Members of ablation sets $\mathcal{A}_{10}$-$\mathcal{A}_6$ ranked by their diffusion time. For each transcript profile the header gives the diffusion time (t_d) and the number of shuffled spots (Shuffled : N).

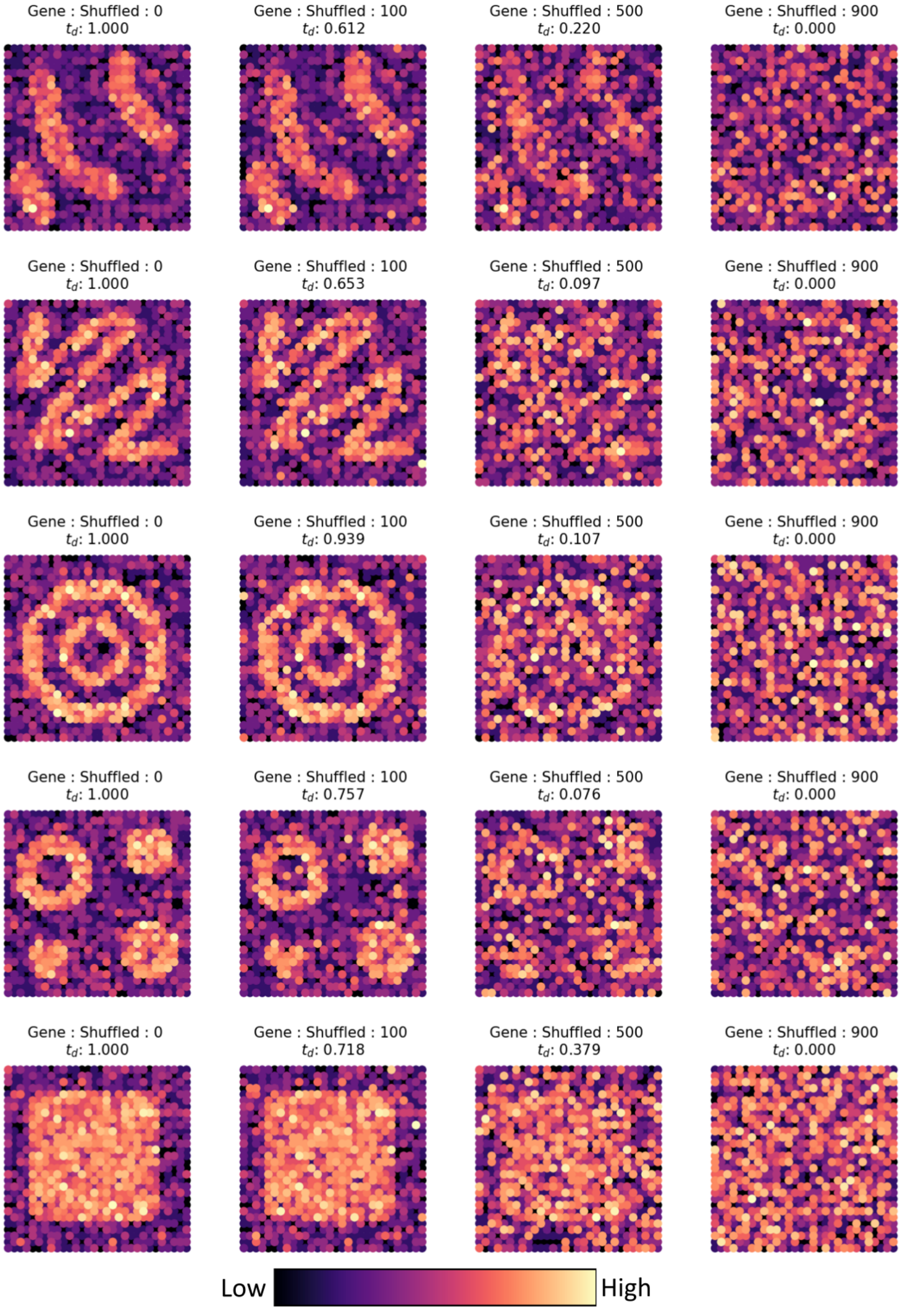

Supplementary Figure 12: Members of ablation sets $\mathcal{A}_5$-$\mathcal{A}_1$ ranked by their diffusion time. For each transcript profile the header gives the diffusion time (t_d) and the number of shuffled spots (Shuffled : N).

S5.4 MOB

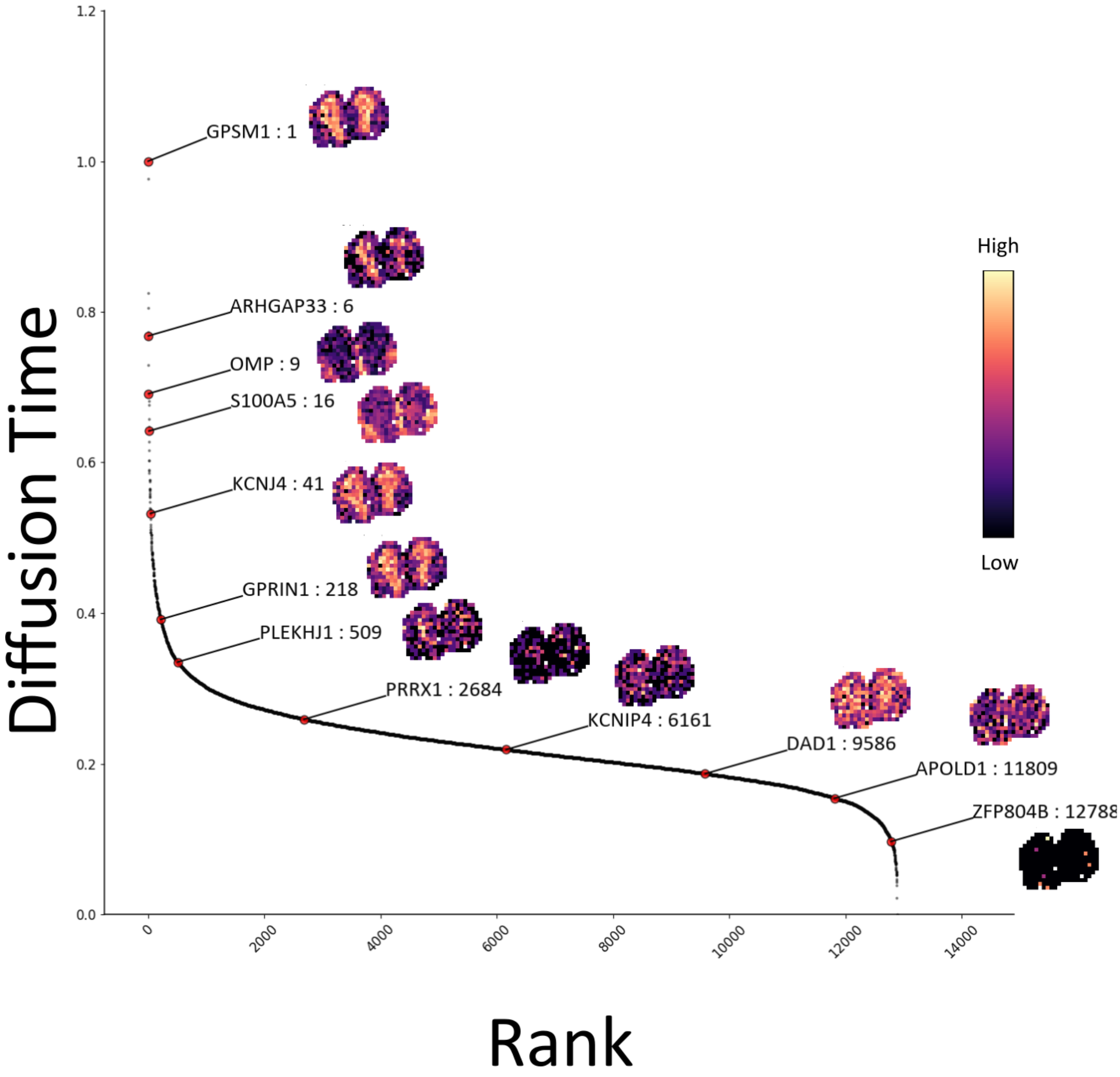

Supplementary Figure 13: Expression profiles along the ranking gradient. Each transcript profile is given in the format Gene : Rank.

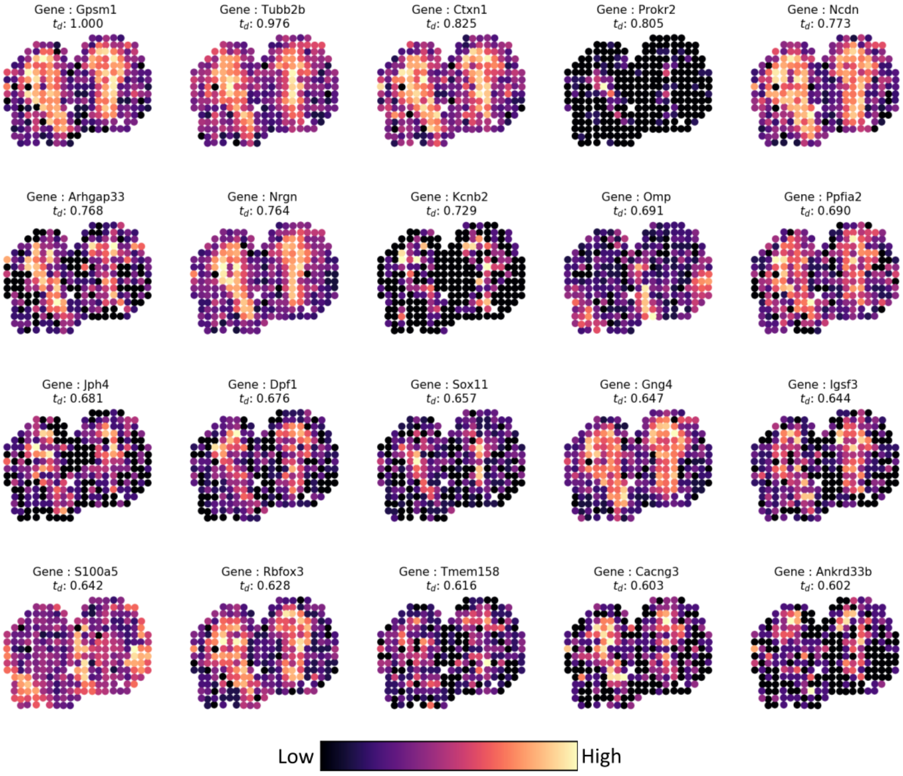

Supplementary Figure 14: Top 20 transcription profiles as ranked by *sepal* for the MOB (ST). For each transcript profile the header gives the diffusion time (t_d) and the name of the associated gene (Gene : X).

S5.5 Mouse Brain

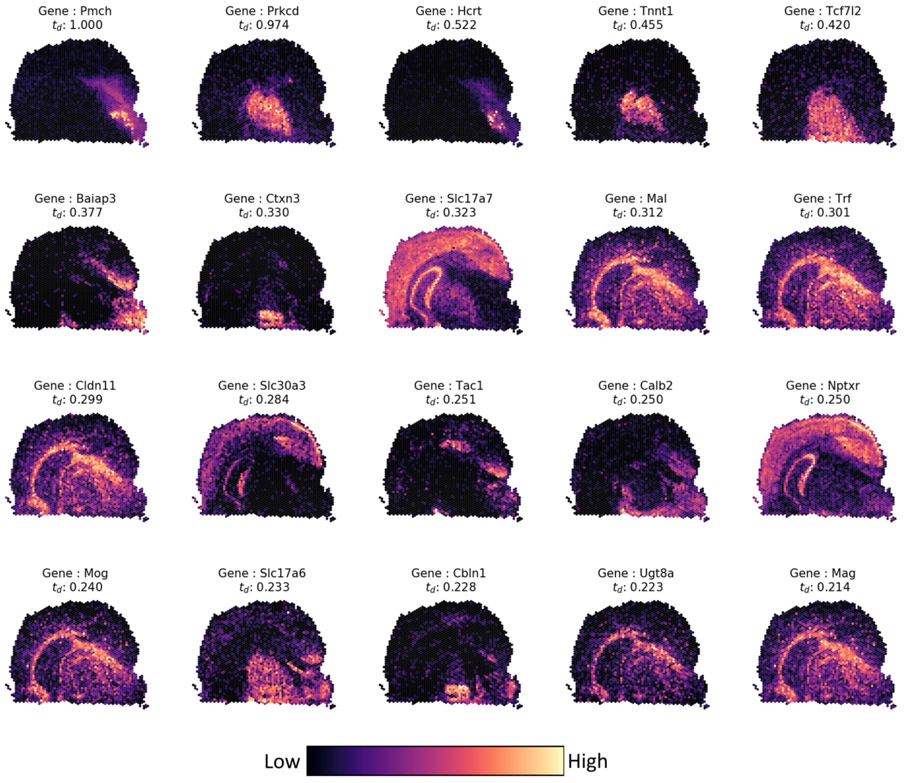

Supplementary Figure 15: Top 20 transcription profiles as ranked by *sepal* for the mouse brain sample (Visium). For each transcript profile the header gives the diffusion time (t_d) and the name of the associated gene (Gene : X).

S5.5.1 Comparison with highly variable genes

To assess how our method to identify *spatially variable genes* compares to standard methods of selecting *highly variable genes* we computed dispersion values (a variance related metric) for the genes in the mouse brain data set using state of the art methods. More specifically we first normalized the data by applying the `scanpy.pp.normalize_per_cell(...,counts_per_cell_after=1e4)` function, followed by a log-transformation using `scanpy.pp.log1p(...)`, finally, dispersion values were obtained by using `scanpy.highly_variable_genes(...,flavor ="seurat")`; `scanpy` version 1.5.1 was used for this analysis. We used the normalized dispersion values ("dispersions_norm") in our comparison. As is illustrated in Supplementary Figure 16, several of the, by *sepal*, top ranked genes are not found in the set of the 1000 most dispersed genes.

From this analysis it becomes clear how only looking at variance-based metrics, not considering spatial information, can lead to transcript profiles with distinct spatial structures being overlooked. The spatially variable, but not highly variable, transcript profiles importantly exhibit spatial structure, i.e., they are not false positives, as can be seen in Supplementary Figure 17 where six of the transcript profiles found among the top 100 highest ranked transcript profiles, but excluded from the set of the 1000 most highly variable ones, are shown.

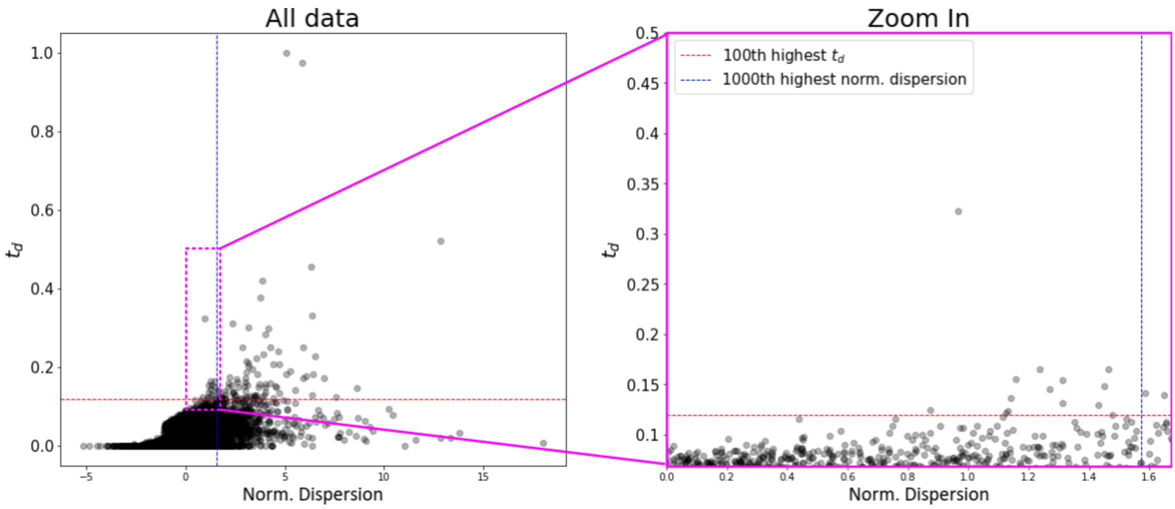

Supplementary Figure 16: For the Mouse Brain data set, diffusion time (t_d) plotted against normalized dispersion values (obtained from `scanpy.pp.highly_variable_genes`). The red dashed line indicate the 100th highest diffusion time, while the blue dashed line indicates the 1000th highest normalize dispersion value. The right pane, with a pink border, is a zoom-in of the marked area in the left pane.

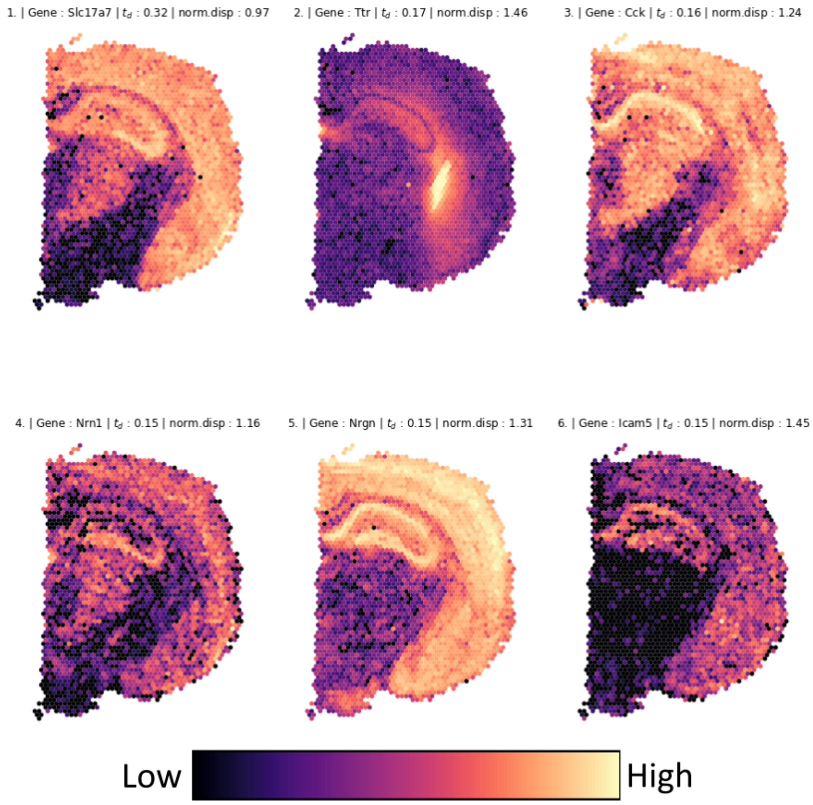

Supplementary Figure 17: For the mouse brain data set, the six transcript profiles with highest diffusion time found among the top 100 highest ranked profiles, by *sepal*, but not present in the set of the 1000 most highly variable ones.

S5.6 Lymph Node

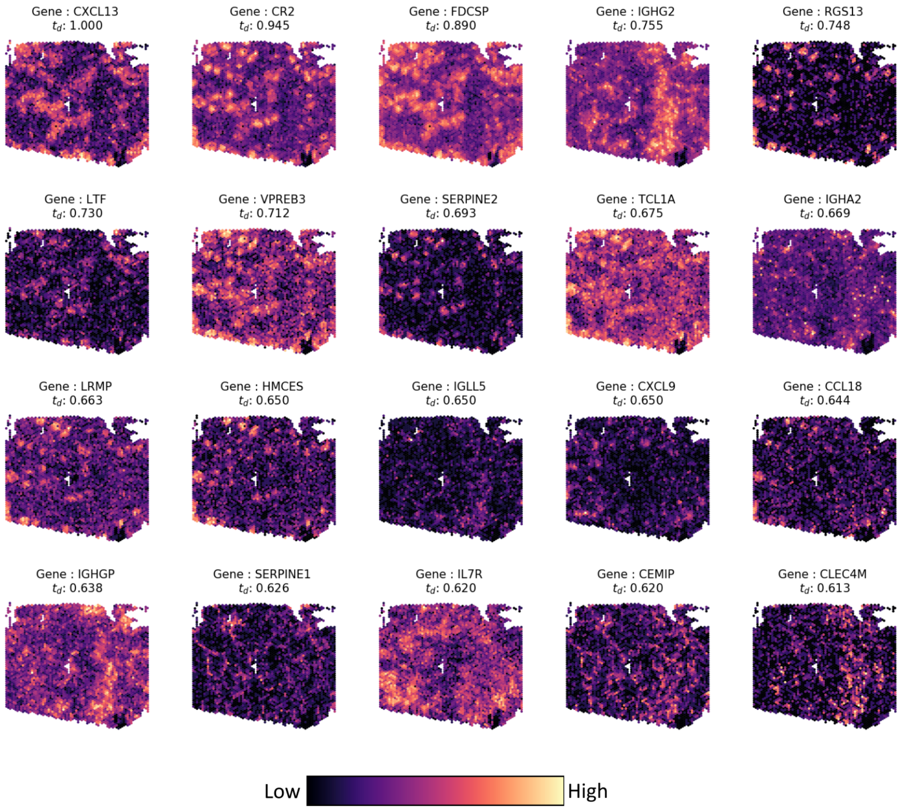

Supplementary Figure 18: Top 20 transcription profiles as ranked by *sepal* for the human lymph node sample (Visium). For each transcript profile the header gives the diffusion time (t_d) and the name of the associated gene (Gene : X).

S5.7 Melanoma

S5.7.1 Top transcription Profiles

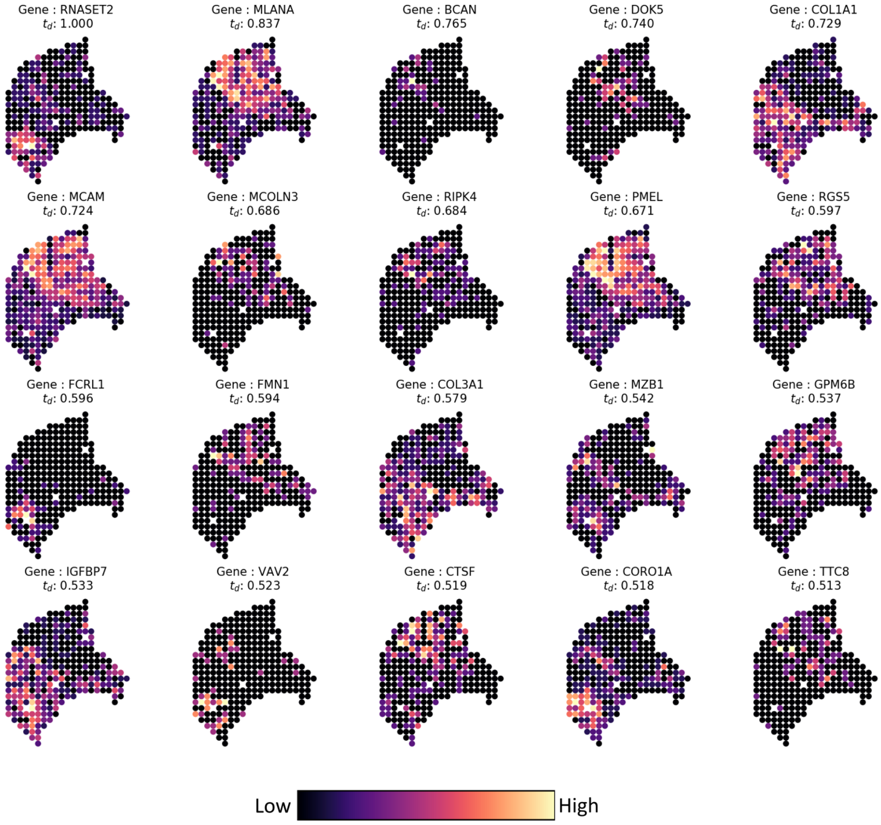

Supplementary Figure 19: Top 20 transcription profiles as ranked by *sepal* for the human melanoma sample (ST 1K). For each transcript profile the header gives the diffusion time (t_d) and the name of the associated gene (Gene : X).

S5.7.2 Representative motifs

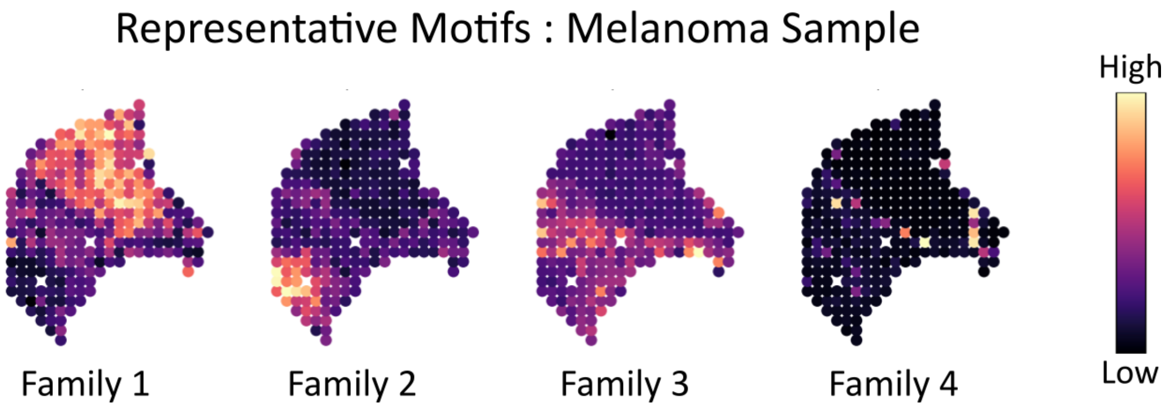

Supplementary Figure 20: Representative patterns for each pattern family identified upon analysis of the melanoma sample.

S5.7.3 Pattern Families

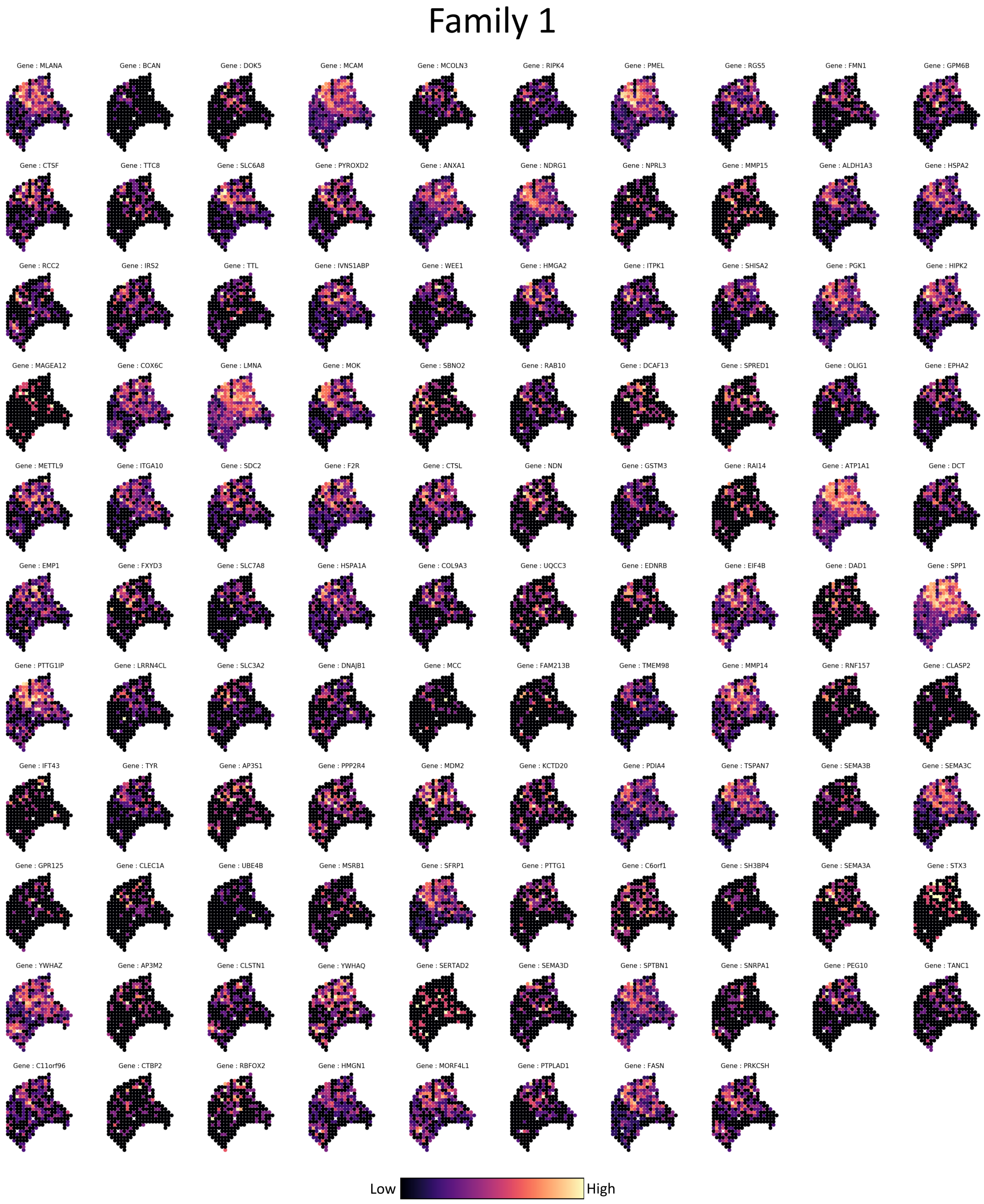

Supplementary Figure 21: All members of pattern family 1 from the melanoma sample. The header of each transcript profile gives the name of the associated gene (Gene : X).

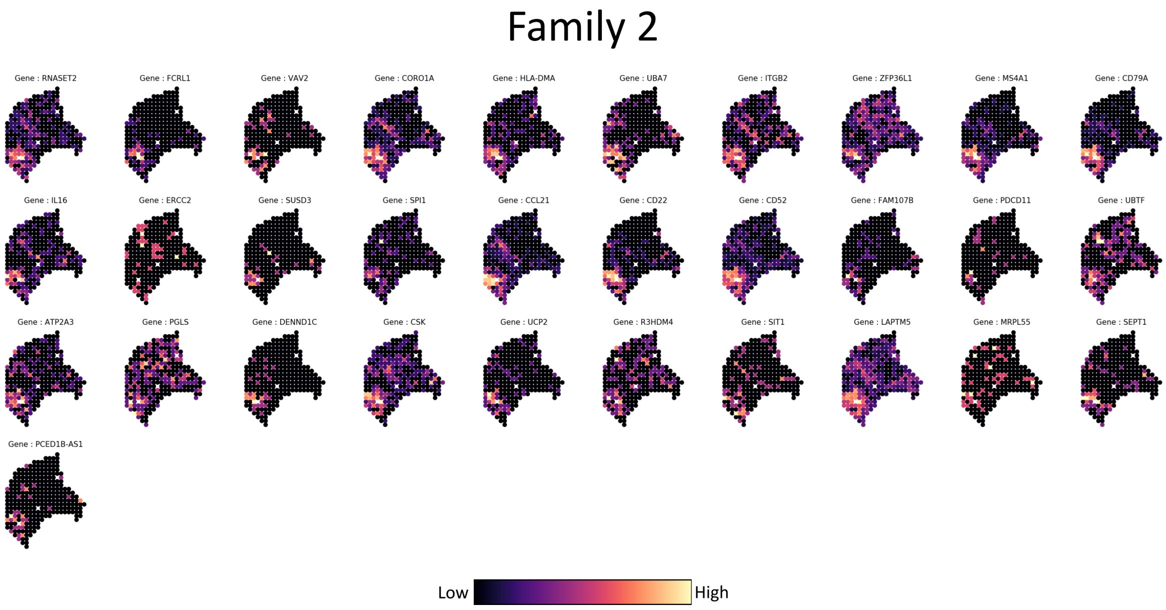

Supplementary Figure 22: All members of pattern family 2. The header of each transcript profile gives the name of the associated gene (Gene : X).

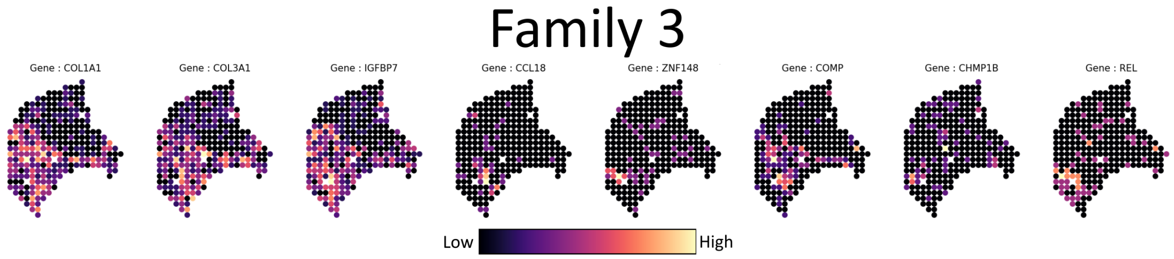

Supplementary Figure 23: All members of pattern family 3. The header of each transcript profile gives the name of the associated gene (Gene : X).

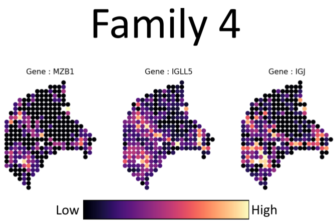

Supplementary Figure 24: All members of pattern family 4. The header of each transcript profile gives the name of the associated gene (Gene : X).

No.	family	native	name	p_value	database	intersection_size
1	1	GO:0007399	nervous system development	5.877865e-08	GO:BP	40
2	1	GO:0022008	neurogenesis	6.382998e-08	GO:BP	33
3	1	GO:0032502	developmental process	6.629987e-08	GO:BP	69
4	1	GO:0048856	anatomical structure development	2.332163e-07	GO:BP	65
5	1	GO:0048699	generation of neurons	1.409125e-06	GO:BP	30
6	1	GO:0030182	neuron differentiation	2.426676e-06	GO:BP	28
7	1	GO:0048666	neuron development	3.545361e-06	GO:BP	25
8	1	GO:0048731	system development	3.997314e-06	GO:BP	56
9	1	GO:0031175	neuron projection development	9.173766e-06	GO:BP	23
10	1	GO:0007275	multicellular organism development	9.470707e-06	GO:BP	59
11	1	GO:0009653	anatomical structure morphogenesis	1.972741e-05	GO:BP	39
12	1	GO:0048468	cell development	2.046510e-05	GO:BP	34
13	1	GO:0030154	cell differentiation	2.246740e-05	GO:BP	50
14	1	GO:0120036	plasma membrane bounded cell projection organi...	7.306454e-05	GO:BP	27
15	1	GO:0009888	tissue development	9.632626e-05	GO:BP	32
16	1	GO:0048869	cellular developmental process	1.042874e-04	GO:BP	50
17	1	GO:0030030	cell projection organization	1.226272e-04	GO:BP	27
18	1	GO:0016049	cell growth	1.623013e-04	GO:BP	15
19	1	GO:0048812	neuron projection morphogenesis	2.611952e-04	GO:BP	17
20	1	GO:0032501	multicellular organismal process	3.315609e-04	GO:BP	69
21	1	GO:0120039	plasma membrane bounded cell projection morpho...	3.497468e-04	GO:BP	17
22	1	GO:0048858	cell projection morphogenesis	3.796596e-04	GO:BP	17
23	1	GO:0032990	cell part morphogenesis	5.673360e-04	GO:BP	17
24	1	GO:0001558	regulation of cell growth	9.261239e-04	GO:BP	13
25	1	GO:0040007	growth	1.080730e-03	GO:BP	20
26	1	GO:0060560	developmental growth involved in morphogenesis	1.524228e-03	GO:BP	10
27	1	GO:0030308	negative regulation of cell growth	1.694435e-03	GO:BP	9
28	1	GO:1901888	regulation of cell junction assembly	1.751998e-03	GO:BP	7
29	1	GO:0040008	regulation of growth	2.081738e-03	GO:BP	16
30	1	GO:0048667	cell morphogenesis involved in neuron differen...	2.184897e-03	GO:BP	15
31	1	GO:0051893	regulation of focal adhesion assembly	2.225958e-03	GO:BP	6
32	1	GO:0090109	regulation of cell-substrate junction assembly	2.225958e-03	GO:BP	6
33	1	GO:0150116	regulation of cell-substrate junction organiza...	2.225958e-03	GO:BP	6
34	1	GO:0016043	cellular component organization	3.014904e-03	GO:BP	59
35	1	GO:0071840	cellular component organization or biogenesis	3.290805e-03	GO:BP	60
36	1	GO:0006928	movement of cell or subcellular component	4.357310e-03	GO:BP	30
37	1	GO:0007409	axonogenesis	4.808180e-03	GO:BP	13
38	1	GO:0051129	negative regulation of cellular component orga...	5.592873e-03	GO:BP	16
39	1	GO:0000904	cell morphogenesis involved in differentiation	7.961997e-03	GO:BP	16
40	1	GO:0048513	animal organ development	8.528914e-03	GO:BP	40
41	1	GO:0000902	cell morphogenesis	9.010142e-03	GO:BP	19
42	1	GO:0032989	cellular component morphogenesis	9.717041e-03	GO:BP	20
43	1	GO:0042221	response to chemical	1.002560e-02	GO:BP	48
44	1	GO:0048041	focal adhesion assembly	1.131274e-02	GO:BP	6
45	1	GO:0061564	axon development	1.229277e-02	GO:BP	13
46	1	GO:0040011	locomotion	1.416100e-02	GO:BP	27
47	1	GO:0048771	tissue remodeling	1.477759e-02	GO:BP	8
48	1	GO:0014033	neural crest cell differentiation	1.588551e-02	GO:BP	6
49	1	GO:0048843	negative regulation of axon extension involved...	1.753755e-02	GO:BP	4
50	1	GO:0051128	regulation of cellular component organization	1.755100e-02	GO:BP	31
51	1	GO:0048523	negative regulation of cellular process	1.811938e-02	GO:BP	48
52	1	GO:0045926	negative regulation of growth	2.045986e-02	GO:BP	9
53	1	GO:0001755	neural crest cell migration	2.052745e-02	GO:BP	5
54	1	GO:1902668	negative regulation of axon guidance	2.412519e-02	GO:BP	4
55	1	GO:2000026	regulation of multicellular organismal develop...	2.760539e-02	GO:BP	28
56	1	GO:0010771	negative regulation of cell morphogenesis invo...	2.951385e-02	GO:BP	6
57	1	GO:0048585	negative regulation of response to stimulus	3.092712e-02	GO:BP	24

58	1	GO:0150115	cell-substrate junction organization	3.127869e-02	GO:BP	6
59	1	GO:0007044	cell-substrate junction assembly	3.127869e-02	GO:BP	6
60	1	GO:0016477	cell migration	3.312975e-02	GO:BP	23
61	1	GO:0030334	regulation of cell migration	4.095770e-02	GO:BP	17
62	1	GO:0010721	negative regulation of cell development	4.114857e-02	GO:BP	10
63	1	GO:0048729	tissue morphogenesis	4.142379e-02	GO:BP	14
64	1	GO:0048841	regulation of axon extension involved in axon ...	4.252604e-02	GO:BP	4
65	1	GO:0060485	mesenchyme development	4.293522e-02	GO:BP	9
66	1	GO:0010977	negative regulation of neuron projection devel...	4.368657e-02	GO:BP	7
67	1	GO:0090084	negative regulation of inclusion body assembly	4.516231e-02	GO:BP	3
68	1	GO:0007411	axon guidance	4.799334e-02	GO:BP	9
69	1	GO:0032879	regulation of localization	4.801707e-02	GO:BP	33
70	2	GO:0046649	lymphocyte activation	4.795319e-06	GO:BP	11
71	2	GO:0001775	cell activation	4.256175e-05	GO:BP	13
72	2	GO:0045321	leukocyte activation	1.272893e-04	GO:BP	12
73	2	GO:0002376	immune system process	1.684961e-03	GO:BP	16
74	2	GO:0002682	regulation of immune system process	1.653754e-02	GO:BP	11
75	2	GO:0002694	regulation of leukocyte activation	2.728215e-02	GO:BP	7
76	2	GO:0050865	regulation of cell activation	4.268626e-02	GO:BP	7
77	2	GO:0048872	homeostasis of number of cells	4.504966e-02	GO:BP	5
78	2	GO:0002684	positive regulation of immune system process	4.670279e-02	GO:BP	9
79	3	GO:0030199	collagen fibril organization	1.654811e-03	GO:BP	3
80	3	GO:0030168	platelet activation	4.127980e-02	GO:BP	3
81	4	GO:0002640	regulation of immunoglobulin biosynthetic process	4.986357e-02	GO:BP	1
82	4	GO:0002642	positive regulation of immunoglobulin biosynth...	4.986357e-02	GO:BP	1

Supplementary Table 3: Result from functional enrichment analysis using g:Profiler (database GP:BP) for each of the identified pattern families. The column ”family” indicate which pattern family that the pathway was enriched within.

S5.8 Mouse Cerebellum

We analyzed a subset of the Slide-seq data, randomly selecting 10000 capture locations, and subdued this subsampled set for analysis using *sepal*. Since the capture locations are do not follow a grid pattern (i.e., it is unstructured) we use the procedure described in S2.1 to augment a structured data set to which our model may be applied. Due to the random character of the capture locations, they may (and do) overlap to some extent, hence we use a different visualization approach for this data. We color each marker blue and let the alpha-level represent the (normalized) observed expression value, rather than using a color gradient. The top 20 genes (w.r.t. diffusion time) are found in Figure 25.

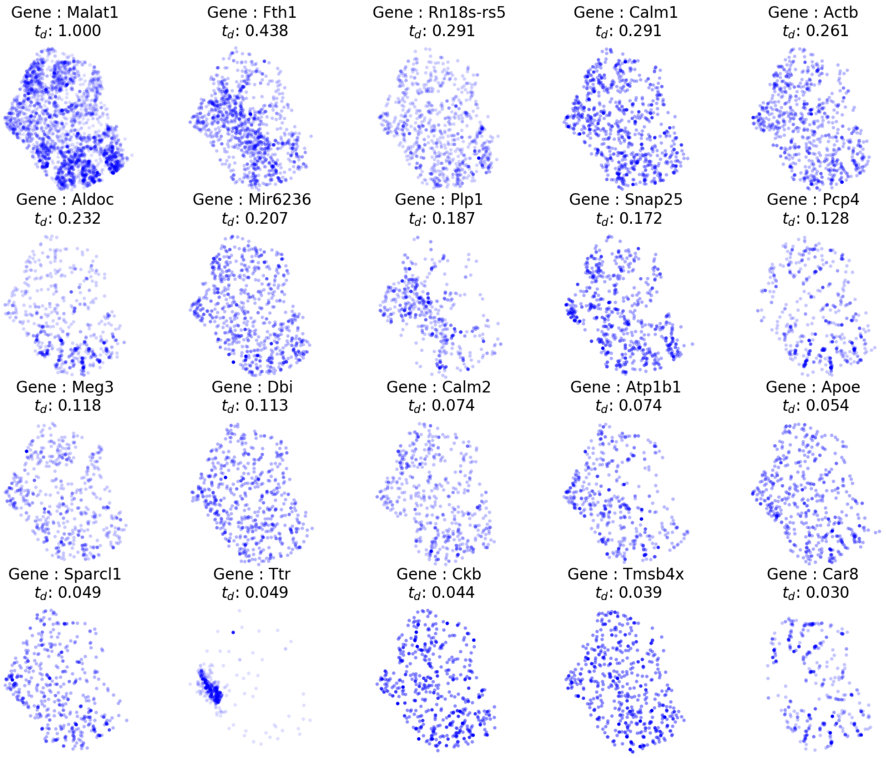

Supplementary Figure 25: Top 20 ranked genes for the mouse cerebellum data set (Slide-seq).

S5.9 Comparison

We first compared the top-ranked (top 20) profiles of each method, to see how these compared and what differences/similarities that could be identified, results are shown in Supplementary Figure 26-27.

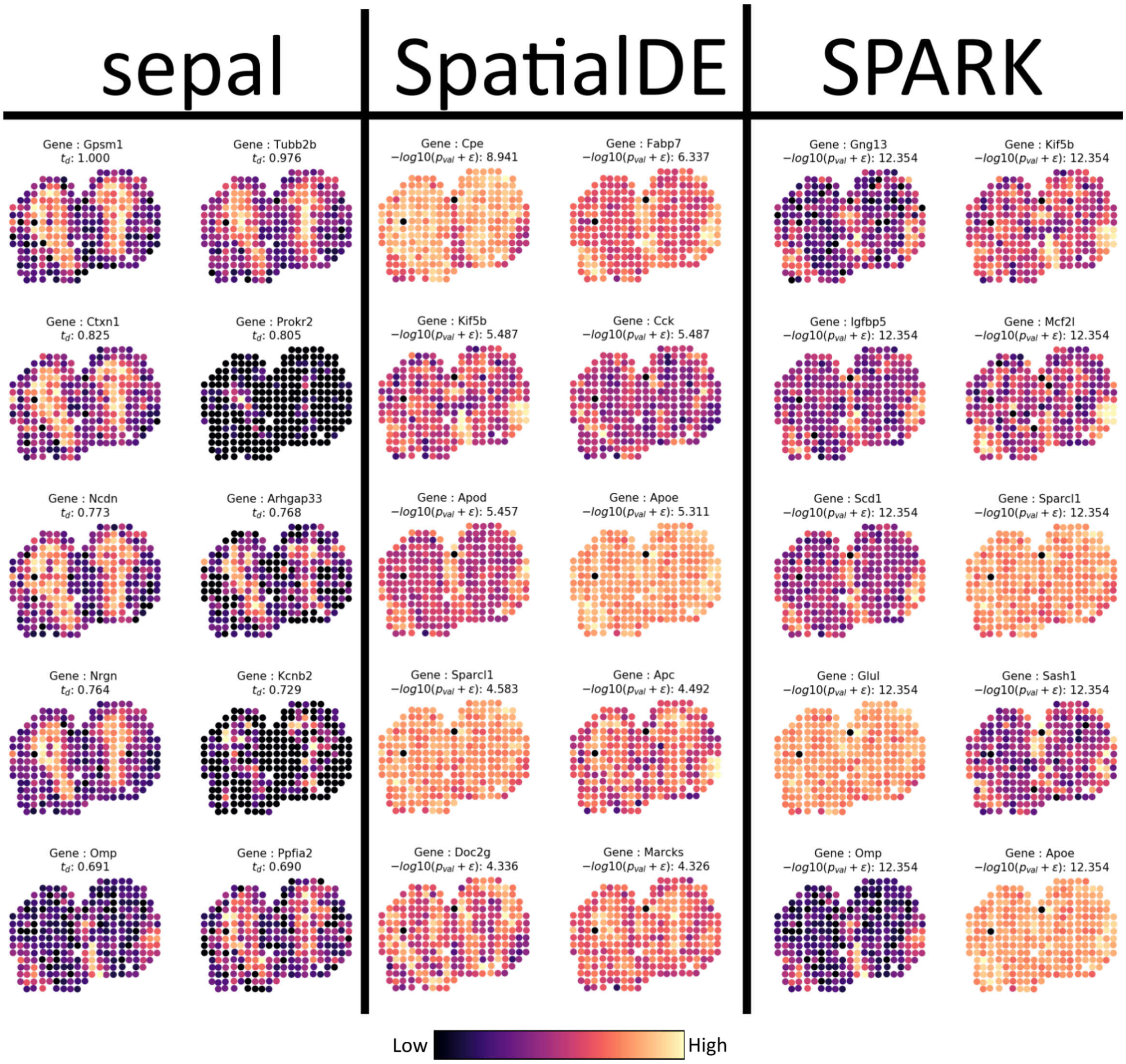

Supplementary Figure 26: Top expression profiles (1 to 10) of the MOB sample, ranked by *sepal*, SpatialDE and SPARK. For *sepal* we give the diffusion time, while for the other two methods we present the the negative logarithm (base 10) of the p-value. A pseudocount of $\epsilon = 10^{-273}$ is added to handle rounded p-values (approximated as zero).

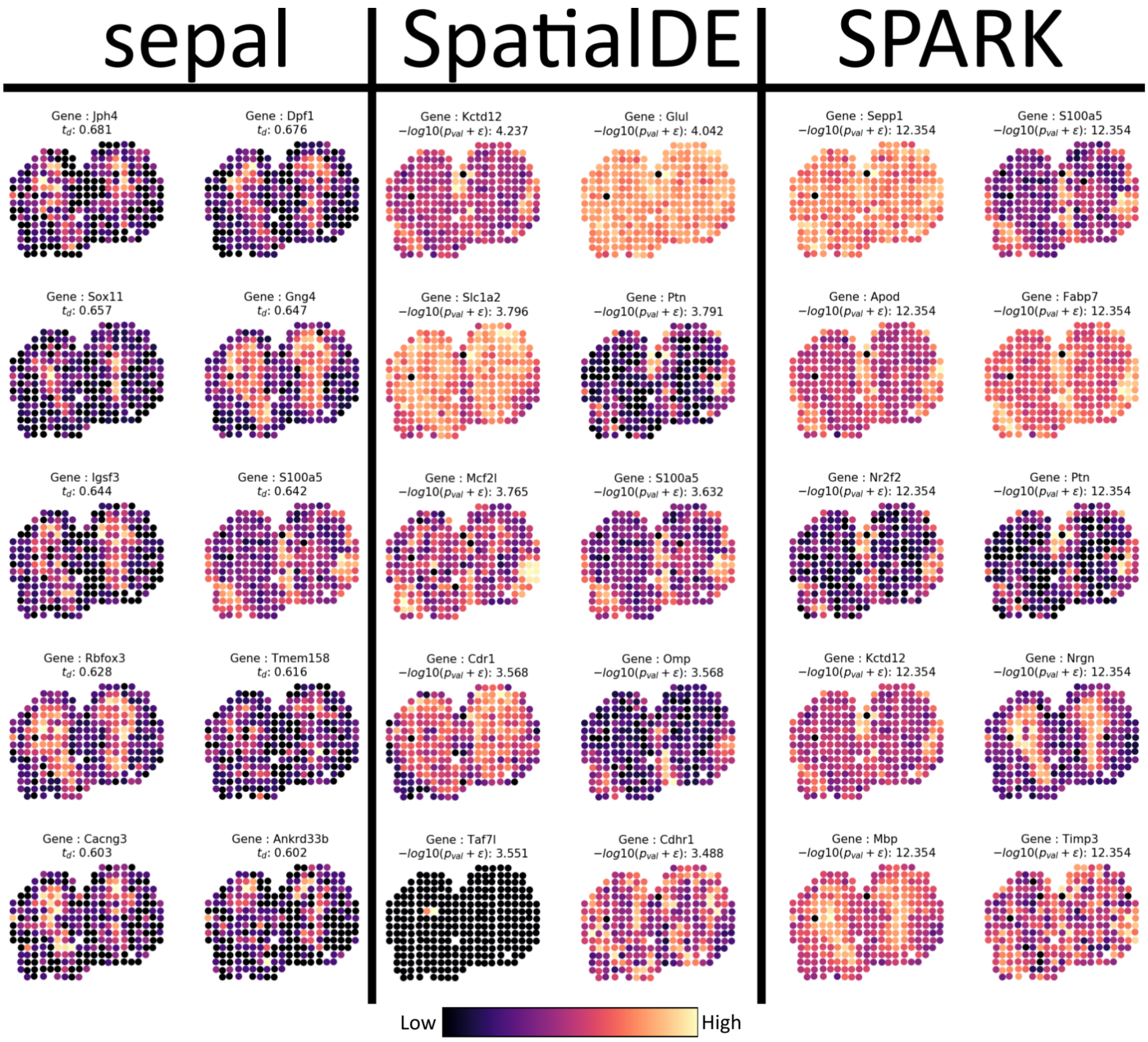

Supplementary Figure 27: Top expression profiles (11 to 20) of the MOB sample, ranked by *sepal*, SpatialDE and SPARK. For *sepal* we give the diffusion time, while for the other two methods we present the the negative logarithm (base 10) of the p-value. A pseudocount of $\epsilon = 10^{-273}$ is added to handle rounded p-values (approximated as zero).

Next, we extend our comparison as to provide a slightly more comprehensive overview of the different type of transcript profiles that each method identified. For each method we extracted the top 100 and 500 highest ranked transcript profiles (using the same ranking system as previously described) and examined how the three sets related to each other, results are visualized as Venn Diagrams in Supplementary Figure 28. In both cases, the number of profiles listed by all three methods ($17 : N = 100$ and $77 : N = 500$) constitute a minority of the profiles. In Supplementary Figures 29-33 we display the transcript profiles exclusively listed by respective method and those profiles listed by all methods in the top 100 case. We can clearly observe diversity in the type of results that respective method present, which emphasizes the value of having multiple different tools to choose from upon any analysis. Inspecting the set of exclusively listed profile, the trend of SpatialDE and SPARK identifying "homogeneous" profiles can again be observed in these larger sets (e.g., *Kcnj10, Epas1, Rcn2,Gja1, Olfm1, Igfb2, Trnp1*), while some of the profiles that *sepal* present are very fragmented (e.g., *Pcgf6, Gfod1 and Rhcg1*) and arguably not always constituting a spatially coherent and structured pattern. The unique patterns from SpatialDE, aside from listing certain homogeneous profiles also has an abundance of very sparse profiles, where a few spots have very strong signal compared to the others (e.g., *Gna14, Filip1, Olfr635, Taf7l, Ddi2, Csf2rb* and *Sfrp5*).

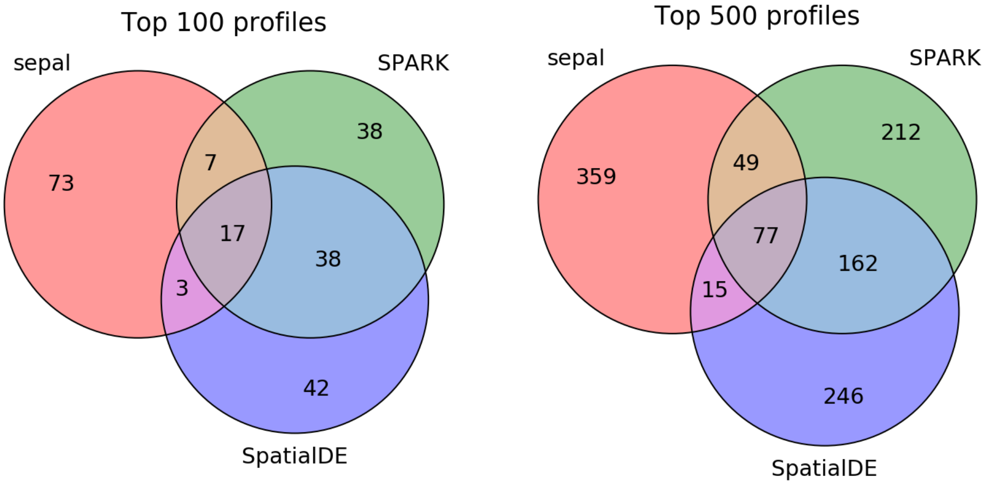

Supplementary Figure 28: Venn diagram of showing the overlap between the top N ranked transcript profiles from respective method (left : $N = 100$, right : $N = 500$).

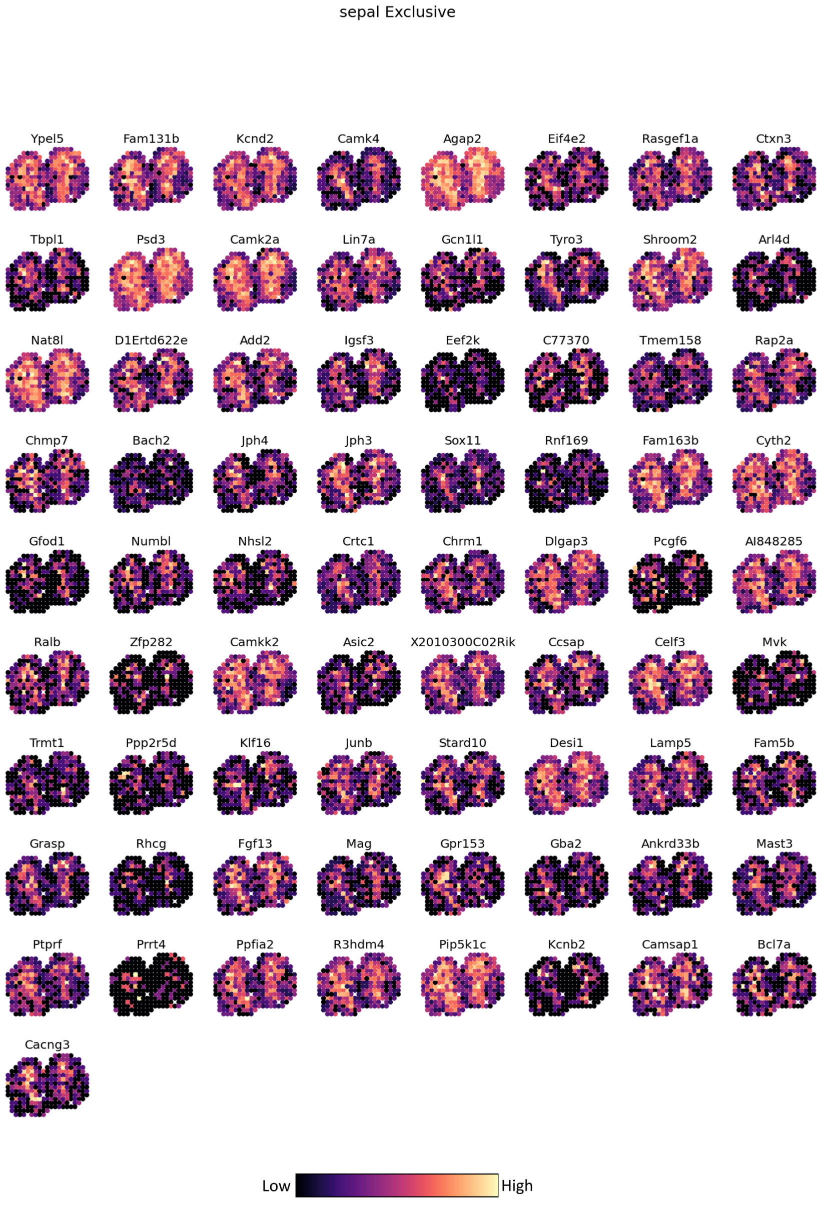

Supplementary Figure 29: Transcript profiles exclusively listed by *sepal* (from $N = 100$).

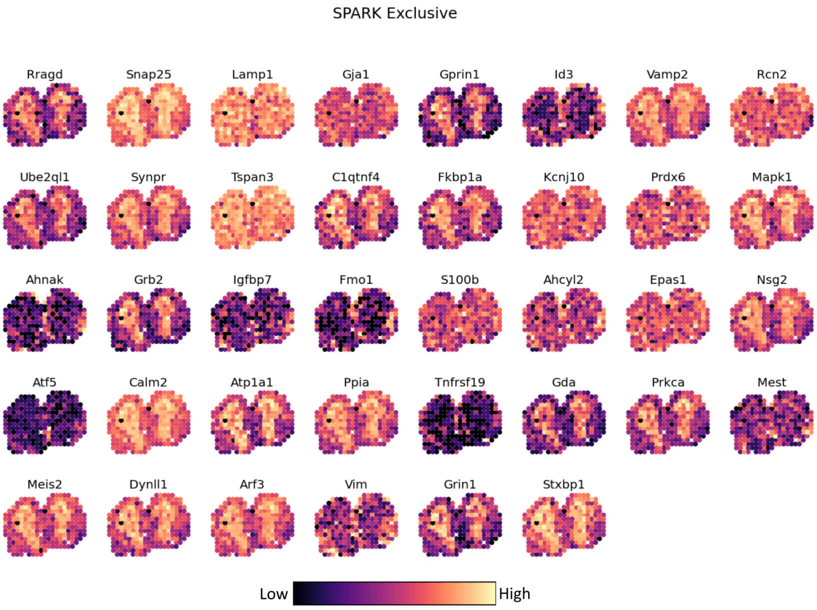

Supplementary Figure 30: Transcript profiles exclusively listed by SPARK (from $N = 100$).

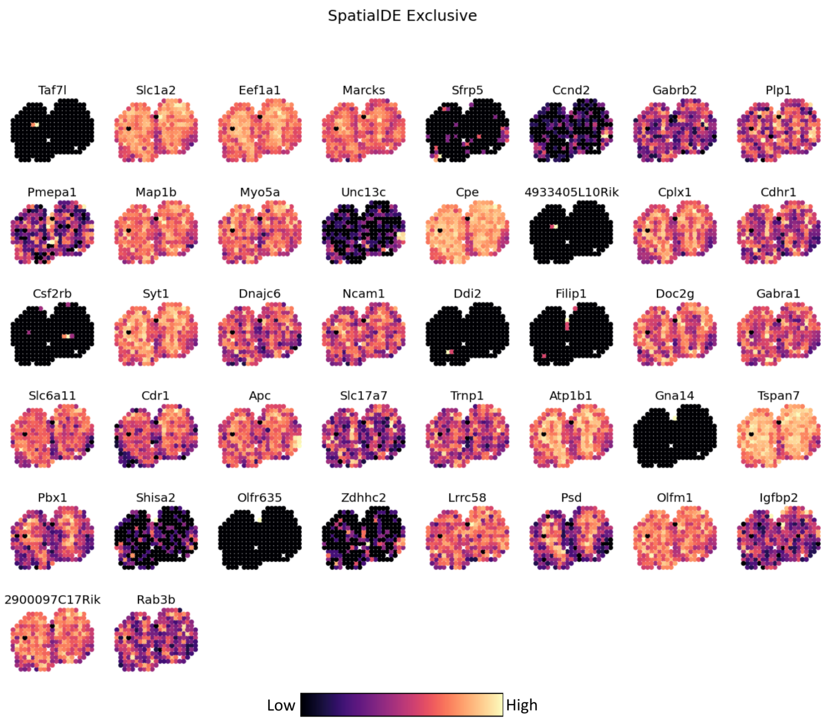

Supplementary Figure 31: Transcript profiles exclusively listed by SpatialDE (from $N = 100$).

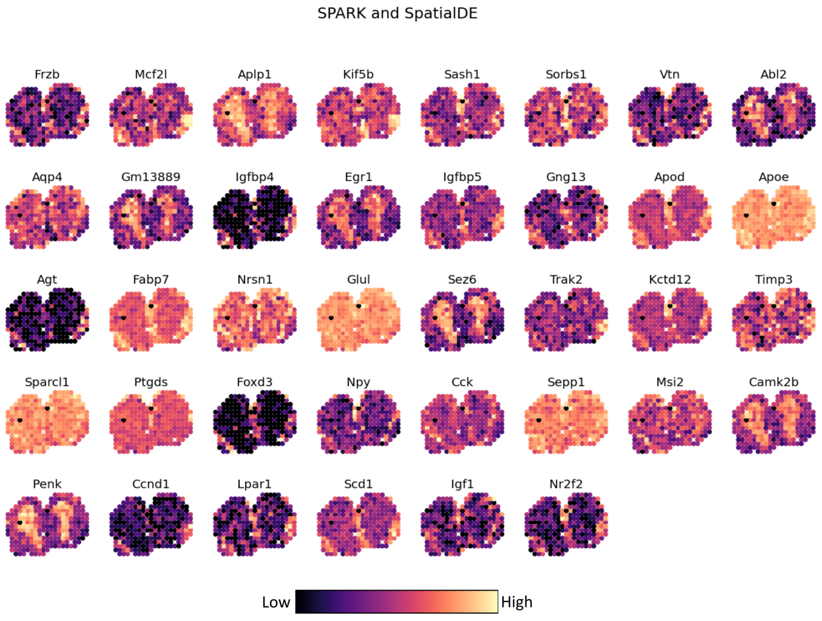

Supplementary Figure 32: Transcript profiles listed by SPARK and SpatialDE (from $N = 100$).

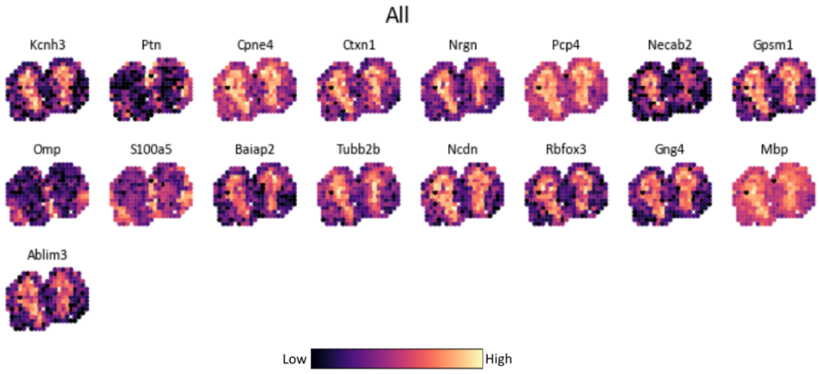

Supplementary Figure 33: Transcript profiles listed by all methods (from $N = 100$).

Finally, we examined how informed by a transcript profile's expression level that respective method's ranking was. This was done using the Spearman correlation, for SpatialDE and SPARK the ranking is based on the q/p-values. The results are shown in Supplementary Table 4.

	sepal	SpatialDE	SPARK
Spearman (ρ)	1.398595e-01	-1.876725e-01	-2.576362e-01 (-2.859163e-01)
p-value	3.561635e-48	6.008244e-86	7.838931e-163 (1.081726e-201)

Supplementary Table 4: Spearman correlation between gene expression level and rank metric (diffusion time and q/p-values respectively) for all three methods included in the comparison; *sepal* SpatialDE, SPARK. Values in parenthesis are computed from the adjusted pvalues for SPARK, with the other based on the combined p-values.

S5.10 Performance Benchmarking

With experimental techniques constantly being refined, the number of observations within each experiment tends to increase, and as such the size of the data sets to be analyzed. While performance rarely is the sole determining factor when choosing a method, it's necessary that results are obtained within a reasonable amount of time, and swift results usually makes the workflow more seamless. Thus, we have examined the performance of ours and the alternative methods.

To put our method into context of those to which we compare it with, we registered the time it took for each method to analyze the MOB sample. The MOB sample was used for the same reason as given for the comparative analysis presented in Supplementary Section S5.9; both alternative methods provide explicit code and/or recommendations regarding how to analyze this sample. We used the same filtering procedure as specified for SpatialDE (minimal number of total gene counts ≥ 3) resulting in a total of 14859 profiles being analyzed. Supplementary Figure 34 and Supplementary Table 5 illustrates the results from this benchmarking.

Method	Cores : 1	Cores : 2	Cores : 3	Cores : 4
sepal	9.425	4.832	3.162	2.197
SPARK	24.156	15.522	13.493	12.539
SpatialDE	7.599	7.527	7.613	7.856

Supplementary Table 5: Performance Benchmarking - Same data as Supplementary Figure 34 but in tabulated form. Rows represent methods, columns analysis with varying number of cores, elements give the time in minutes to complete the analysis, values are rounded to three decimals.

To ensure a fair evaluation, we compared our results with those reported by the authors of SPARK (SPARK publication, Supplementary Table 3) and noted that our times were of similar magnitudes

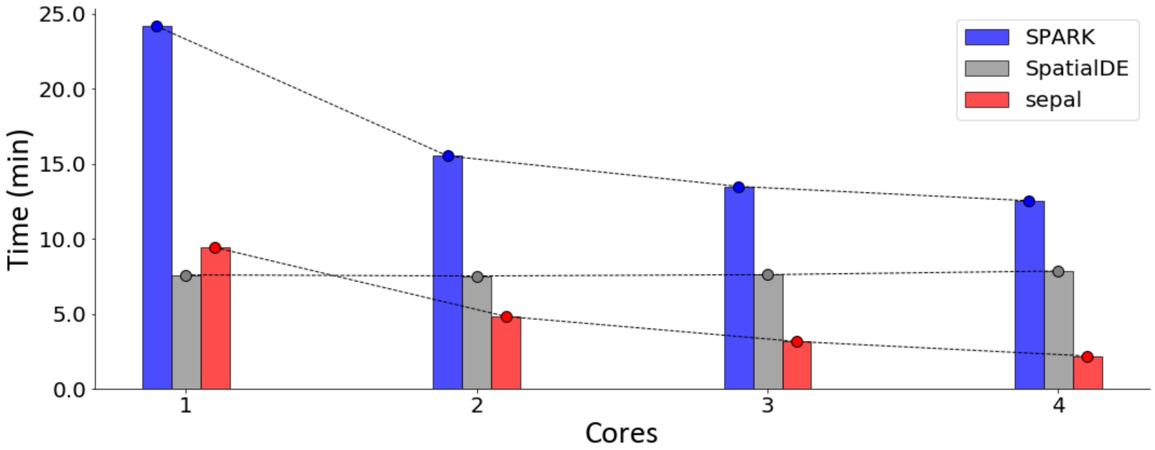

Supplementary Figure 34: Performance Benchmarking - y-axis gives the time in minutes to complete analysis of the 14859 profiles found within the MOB dataset after filtration. The x-axis gives the number of cores (2 threads per core) used in respective analysis. Results for SPARK are shown in blue, SpatialDE in gray and *sepal* in red.

to those reported for the MOB sample. More explicitly the reported times were (using 11274 genes): 46.53min on 1 thread and 5.62min on 10 threads using SPARK, 6.98min on one thread using SpatialDE).[1] As can be seen from the results, *sepal* requires less time to complete the analysis compared to the other two methods in all cases except one (1 Core). As most machines tend to have more than a single core, this implies a faster runtime for *sepal* in general.

Technical Specifications

All analyses were run on a Lenovo ThinkPad P51 20HH0015MX, with a 6th Generation Intel® Core™ i7-6820HQ Processor, which has 4 hyperthreaded cores (8 threads). The operating system was Fedora 29 (LSB version: core-4.1-amd64:core-4.1-noarch). To control the number of cores available for each analysis, we used the `taskset` command (from the `util-linux-ng` package) with the flag `--cpu-list` to specify which (logical) CPUs that should be used; having restricted CPU-accessibility on a system level, we let the methods use all available CPU resources.
